# Co-Development of a Web Application (COVID-19 Social Site) for Long-Term Care Workers (“Something for Us”): User-Centered Design and Participatory Research Study

**DOI:** 10.2196/38359

**Published:** 2022-09-22

**Authors:** Catherine H Saunders, Ailyn Sierpe, Gabrielle Stevens, Glyn Elwyn, Matthew Cantrell, Jaclyn Engel, Melissa Gonzalez, Martha Hayward, Joellen Huebner, Lisa Johnson, Alejandro Jimenez, Nancy Ruth Little, Corinne McKenna, Manu Onteeru, May Oo Khine, Jacqueline Pogue, José Luis Salinas Vargas, Peter Schmidt, Rachael Thomeer, CONFIDENT Study Long-Term Care Partners, Marie-Anne Durand

**Affiliations:** 1 The Dartmouth Institute for Health Policy & Clinical Practice Dartmouth College Lebanon, NH United States; 2 National Association of Health Care Assistants Carl Junction, MO United States; 3 Institute for Healthcare Improvement Boston, MA United States; 4 Yellow Lion Media New York City, NY United States; 5 Department of Public Health East Carolina University Greenville, NC United States; 6 Department of Neurology NYU Grossman School of Medicine New York City, NY United States; 7 Centre for Epidemiology and Research in Population Health Université de Toulouse Toulouse France

**Keywords:** COVID-19, vaccine hesitancy, long-term care, social media, web application, website, intervention development, information and communications technology

## Abstract

**Background:**

Improving confidence in and uptake of COVID-19 vaccines and boosters among long-term care workers (LTCWs) is a crucial public health goal, given their role in the care of elderly people and people at risk. While difficult to reach with workplace communication interventions, most LTCWs regularly use social media and smartphones. Various social media interventions have improved attitudes and uptake for other vaccines and hold promise for the LTCW population.

**Objective:**

We aimed to develop a curated social web application (interactive website) to increase COVID-19 vaccine confidence (a 3-arm randomized trial is underway).

**Methods:**

Following user-centric design and participatory research approaches, we undertook the following 3 steps: (1) content identification, (2) platform development, and (3) community building. A LTCW and stakeholder advisory group provided iterative input. For content identification (step 1), we identified topics of concern about COVID-19 vaccines via desktop research (published literature, public opinion polls, and social media monitoring), refined by interviewing and polling LTCWs. We also conducted a national online panel survey. We curated and fact-checked posts from popular social media platforms that addressed the identified concerns. During platform development (step 2), we solicited preferences for design and functionality via interviews and user experience testing with LTCWs. We also identified best practices for online community building (step 3).

**Results:**

In the interviews (n=9), we identified 3 themes: (1) LTCWs are proud of their work but feel undervalued; (2) LTCWs have varying levels of trust in COVID-19–related information; and (3) LTCWs would welcome a curated COVID-19 resource that is easy to understand and use-"something for us". Through desktop research, LTCW interviews, and our national online panel survey (n=592) we found that participants are interested in information about COVID-19 in general, vaccine benefits, vaccine risks, and vaccine development. Content identification resulted in 434 posts addressing these topic areas, with 209 uploaded to the final web application. Our LTCW poll (n=8) revealed preferences for personal stories and video content. The platform we developed is an accessible WordPress-based social media web application, refined through formal (n=3) and informal user experience testing. Users can sort posts by topic or subtopic and react to or comment on posts. To build an online community, we recruited 3 LTCW “community ambassadors” and instructed them to encourage discussion, acknowledge concerns, and offer factual information on COVID-19 vaccines. We also set “community standards” for the web application.

**Conclusions:**

An iterative, user-centric, participatory approach led to the launch of an accessible social media web application with curated content for COVID-19 vaccines targeting LTCWs in the United States. Through our trial, we will determine if this approach successfully improves vaccine confidence. If so, a similar social media resource could be used to develop curated social media interventions in other populations and with other public health goals.

## Introduction

COVID-19 vaccination rates among long-term care workers (LTCWs) vary across the United States, partly due to a patchwork of legal challenges to a Centers for Medicare and Medicaid Services vaccination mandate [1]. Full vaccination rates range from 70% to 99%, with booster rates trailing from 17% to 56% [2].

Long-term care settings have been major outbreak sites throughout the COVID-19 pandemic, leading to illness and death among vulnerable residents and staff. Researchers of the Centers for Disease Control and Prevention estimate that more than 2300 LTCWs and 151,000 residents have died from COVID-19. COVID-19 outbreaks threaten LTCWs themselves and their often underserved communities [3]. More than half of LTCWs are from disadvantaged socioeconomic, racial, or ethnic groups [4]. LTCWs from underserved communities and those with lower educational attainment are less likely to be vaccinated for COVID-19 than those from advantaged backgrounds [5]. Long-term care staff shortages predate the pandemic, but recent data from the Bureau of Labor Statistics show a further loss of nearly 400,000 LTCWs from 2020 to 2021 [6,7].

Because LTCWs care for the frailest elderly, increasing vaccine confidence and uptake in this population while simultaneously supporting and retaining the LTCW workforce is a critical public health need.

Although improving vaccine confidence and uptake among LTCWs is challenging, social media is a promising potential solution. Social media–based interventions can suit marginalized groups and LTCWs already relying on social media for information (personal communication, Matthew Cantrell, April 2021)[8]. Randomized trials of social media interventions have shown some success, largely in improving attitudes about other vaccines [9-12]. When combined with selected information from medical experts and communication between participants, they have also improved vaccine uptake [10,13]. As far as we know, no social media web applications target LTCWs and address their questions and concerns about COVID-19 vaccines. It is unknown whether this type of intervention would improve confidence or uptake of COVID-19 vaccines and boosters.

We aimed to fill this gap by developing a curated social media web application for LTCWs with low confidence in COVID-19 vaccines.

## Methods

### Study Design

We conducted a user-centered design and participatory research study to develop, qualitatively assess (usability and acceptability), and deploy a social media web application (interactive website) called the COVID-19 Social Site [14,15]. We curated and customized the site for LTCWs with low COVID-19 vaccine confidence within a broader randomized clinical trial (ClinicalTrials.gov, NCT05168800), funded by the Patient-Centered Outcomes Research Institute (COVID-2021C2-13181).

We reported results using the Consolidated Criteria for Reporting Qualitative Research (CORE-Q) and the Checklist for Reporting Results of Internet E-Surveys (CHERRIES) [16,17].

### Participatory Approach

Our National Association of Health Care Assistants (NAHCA) partners were critical to web application development. Their deep expertise in long-term care informed development from conception to launch.

We recruited 10 LTCW partners from diverse backgrounds and positions as part of a stakeholder advisory group, which met regularly. Our partners were instrumental in designing and developing the web app, and providing iterative feedback and advice throughout all stages of the project. Their feedback is captured throughout this manuscript (Multimedia Appendix 1).

### Theoretical Framework and Context

We conceived this intervention within a broader comparative effectiveness trial with 2 interventions guided by the theoretical framework by Peretti-Watel et al for vaccine hesitancy, which considers vaccine hesitancy a decision-making process, not a static state [18,19]. Therefore, interventions that increase knowledge in the right context could also increase vaccine confidence and uptake [20]. Emerging evidence suggests that multi-component dialogue-based interventions can be effective, particularly when context and hesitancy drivers are taken into account. Developers must also tailor content, format, and delivery to specific audiences [20].

### Ethics Approval

Dartmouth College’s Committee for the Protection of Human Subjects approved this study (STUDY00032340).

### Step 1. Content Identification

#### Desktop Research

We first identified the common questions and concerns associated with low COVID-19 vaccine confidence by reviewing information from the published literature, public opinion polls, and social media. Given the evolving pandemic, we had a flexible search strategy (Multimedia Appendix 2).

We developed a dynamic list of questions and concerns by consulting with our team (including LTCW partners and other stakeholders) and cross-checking with existing resources [21]. We grouped the questions and concerns into top-level topics. We refined topic wording with plain language principles [22].

#### LTCW Stakeholder Consultation

We shared our top-level topics with our LTCW stakeholder advisors via semistructured interviews. We have provided details on the interview population, recruitment, procedures, and analysis (Multimedia Appendix 3 [22,23]), and the interview guide (Multimedia Appendix 4)[23,24].

We polled our LTCW partners to assess the content mix they wanted on the web application, including questions about the source (platform and creator), type, quantity, and tone of posts. Our questionnaire is presented in Multimedia Appendix 5.

#### National Online Panel Survey

We deployed a Qualtrics (Seattle, WA) survey (Multimedia Appendix 6) using members of an existing panel to gain insights from a model population on the perceived importance of different COVID-19 vaccine-related information. Multimedia Appendix 7 [25-32] provides further details about survey development, sampling, recruitment, and analysis [4,25,26].

#### Content Curation

We sourced material from popular social media web applications according to the preferences identified by LTCWs via interviews and a poll. We plan to continue sourcing content (Multimedia Appendix 2) throughout the life of the site.

#### Content Processing and Fact-Checking

The content team identified social media posts of interest and logged them along with basic details (eg, date posted, the platform of origin, and engagement metrics). We used a category-based system with hashtags.

We designed a fact-checking process (Multimedia Appendix 8) in consultation with our broader advisory group, including LTCW partners and other stakeholders. The study team also reviewed each post to confirm appropriateness in light of content mix preferences.

### Step 2. Platform Development

#### LTCW Stakeholder Consultation

We presented our initial concept for the web application to our LTCW stakeholders, soliciting information about the desired look, feel, and functionality iteratively and during the semistructured interviews mentioned in Step 1. We have provided details on the methods in Multimedia Appendix 3.

#### Web Application Wireframes and Initial Build

We shared our initial concept informed by stakeholder insight with a web design and development company. They advised on the functionality, and look and feel of the web application. The web team developed initial wireframes and the preliminary site on WordPress, primarily using Blade, Javascript, Hypertext Preprocessor (PHP), and Sassy Cascading Style Sheets (SCSS) scripting languages. We iteratively modified and tested the site with the study team, LTCW partners, and other stakeholders.

#### User Experience Testing

After testing and modifying initial wireframes with LTCW partners and other stakeholders, we conducted one-on-one user experience (UX) and user interface (UI) testing sessions with LTCWs who were naive to the study and its goals. We also solicited UX and UI feedback from LTCW partners and other stakeholders during meetings. Multimedia Appendix 9 [26, 33-37] provides more details on our testing approach, including our affinity mapping analysis [33-38].

### Step 3. Community Building

#### LTCW Stakeholder Consultation

During interviews and stakeholder meetings, we asked LTCWs how they wanted the web application to operate and what kind of moderation or guidance we should use. We reviewed the community standards of popular Facebook groups focusing on COVID-19 vaccine discussion. Further details on our approach to community building are provided in Multimedia Appendix 10 [21].

## Results

### Participatory Approach

LTCWs were integral to every stage of this web application development project. Our LTCW partners noted:

Certified nursing assistants are often overlooked and dismissed. We help individuals every day, but it is an honor to be given the opportunity to help so many people on such a large scale.

LTCW stakeholder

To have my opinion considered and appreciated gives me great satisfaction and encourages me to continue empowering my profession.

LTCW stakeholder

### Step 1. Content Identification

#### Desktop Research

We grouped questions and concerns about COVID-19, and COVID-19 vaccines and boosters into the following 5 general topics: access and process, benefits and efficacy, side effects and harm, development process, and the pandemic overall.

#### LTCW Stakeholder Consultation

We interviewed 9 LTCWs (5 certified nursing assistants, 1 food services worker, 1 activities director, 1 maintenance worker, and 1 other direct care worker) working in skilled nursing facilities, home care, and hospice care across various regions in the United States. The LTCWs interviewed included 5 females and 4 males. Four participants were White, 2 were Asian, 2 were Black, and 1 was biracial. All LTCWs indicated English as their preferred language, except for 1 who selected Cantonese. Interviews lasted approximately an hour and occurred over Zoom (Zoom Video Communications) between September 16 and November 5, 2021.

**Figure 1:**
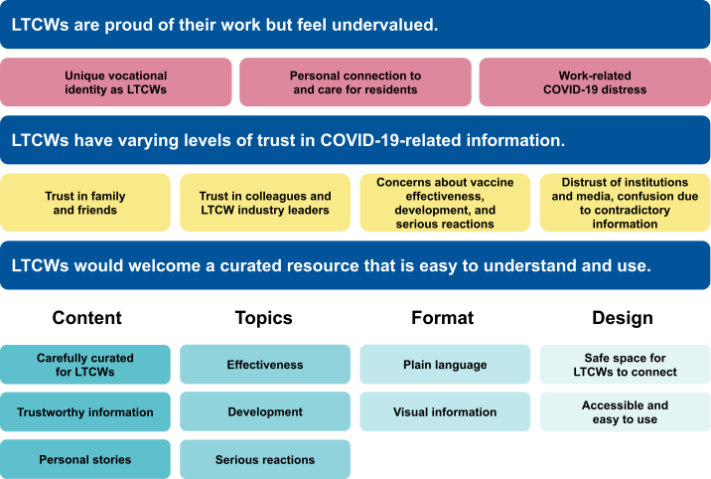
Long-term care worker interview themes. LTCW: long-term care workers.

We found 3 major themes (Figure 1; Multimedia Appendix 1).

##### Theme 1. LTCWs are Proud of Their Work and Yet Feel Undervalued

All interviewees expressed pride and unique vocational identity as LTCWs. They spoke impassionedly, with phrases like

Where my heart is.

Participant #6

I don’t call it my passion, I don’t call it my calling; it’s my ministry.

Participant #4

A minority of participants noted frustration that LTCWs are generally unrecognized in favor of nursing or other health care workers.

LTCWs spontaneously cited their care for and connection to long-term care residents as central to their professional identities.

I love that it’s such a sense of family when we’re there.

Participant #2

This sense of responsibility was a powerful motivator for the LTCWs who decided to get COVID-19 vaccines.

In my line of work, I work with the demographic most at risk [so] I have a moral responsibility to other people.

Participant #2

The web of professional and emotional connections magnified the loss and trauma LTCWs experienced during the pandemic, with most noting death and illness among their residents, colleagues, families, and communities. One participant made the following statement:

Seeing these people that I work with – and I love – [...] in a very quick amount of time go from a healthy senior to gone was very devastating.

Participant #2

Another participant recounted their experience of near hospitalization due to COVID-19, which prompted them to get vaccinated.

##### Theme 2. LTCWs Have Varying Levels of Trust in Information About COVID-19, and COVID-19 Vaccines and Boosters

During the COVID-19 pandemic, LTCWs received information from various sources, including their employers, families and friends, the government, and news and social media. Participants were more likely to trust their families and friends about COVID-19, and its vaccines and boosters than other sources. Sometimes these influences resulted in vaccination, and other times they increased hesitancy. One participant made the following statement:

[My husband] is the one that is hesitant [about] the vaccine, and I am with him. He is the one that influenced me not to get it right now.

Participant #3

LTCWs noted their unique access to up-to-date information, citing the medical directors at their facilities and industry leaders as strong influences. Interviewees viewed most other mainstream information sources, including the government and the news media, as unreliable. One participant made the following statement:

I am not a scientist or a doctor. It’s very hard to find information that’s accurate.

Participant #2

In part because of their mixed information sources and partly due to the contradictory nature of official COVID-19 messages, LTCWs have outstanding questions about COVID-19 vaccines. One participant made the following statement:

Even the CDC and the FDA [...] still have different voices about the vaccine.

Participant #6

Another encapsulated LTCWs’ concerns with the following statement:

It’s [...] still a trial. They do not know 100% the consequences, the side effects, long-term side effects, [...] the ingredients.

Participant #9

Of particular concern were vaccine effectiveness, the vaccine development process, and potential harms, including long-term effects (fertility and unknown future problems) and serious reactions (myocarditis and blood clots). One participant recalled a colleague who made the following statement:

Her only reason was because she didn’t want to end up on a commercial 10 years from now that said, “Have you developed this, this, and this and this because of the [...] COVID vaccine that was forced on you 10 years ago? You may be liable for a lawsuit [sic]”

Participant #7

##### Theme 3. LTCWs Would Welcome a Carefully Curated, Easy-to-Understand and Use COVID-19 Resource

Outstanding questions about COVID-19 and vaccines meant most LTCWs wanted a dedicated place where they could find trustworthy information.

Targeted at who you're trying to get

Participant #4

Knowing they could trust the information was critical.

Just not knowing how to find out, not knowing how to research, or not knowing how to look into the resources. I think it’s really important that be made easily and readily available to people.

Participant #2

LTCW participants said the best way to get curated and trustworthy information was through personal stories and plain clear communication. One participant made the following statement:

Having actual stories to all of this kind of stuff is going to probably be key. People need to connect with other people.

Participant #5

Clear simple communication is paramount, especially given the busy nature of LTCWs’ lives. One interviewee said they needed the following:

Solid information, but in a simpler way, like easy to understand.

Participant #1

Other participants preferred visual information.

I think pictures speak louder than words to a lot of people, especially with social media.

Participant #7

##### Content Topics

Concerning the web application content itself, our LTCW interviewees appreciated the 5 proposed topics but suggested we eliminate the access and process topic. It was clear to LTCWs where and how they could get vaccinated. Additionally, they emphasized the importance of including content about COVID-19 vaccine effectiveness, the development process, and the potential short- and long-term risks. They also worked with us to refine the topic names for clarity (Multimedia Appendix 2). Finally, our stakeholder partners expressed interest in light, non-COVID, LTCW-related topics, tapping into their professional identity.

##### Content Mix

When asked about the desired characteristics of the COVID-19 Social Site’s content, we found that the LTCW stakeholders wanted overall diversity in post format. Infographics and text-based content were most and least favored, respectively. The group strongly preferred content from Facebook and YouTube, with TikTok as the least preferred. They favored posts from certified nursing assistants, scientists, and laypeople and disfavored content from journalists. The LTCW stakeholders slightly preferred serious content over lighthearted content. Finally, they slightly preferred evergreen content to content covering new developments. Additional details are provided in Multimedia Appendix 5.

#### National Online Panel Survey

A total of 592 participants comprised the final survey sample. Participant flow and characteristics are detailed in Multimedia Appendix 7.

A large proportion of participants rated each information topic as at least “a little” important (Table 1; Multimedia Appendix 6). How effectively the vaccines protect people from the virus was most frequently the most important category among respondents. Participants least frequently selected vaccine benefits as the most important category. The other 4 categories were closely grouped. No new topics meaningfully different from the existing topics emerged from open-text responses with sufficient frequency to include. Additional results are provided in Multimedia Appendix 7.

**Table 1:** Ranked importance of COVID-19 topics in a national online panel survey.

Topic rank	Ratings of “a little important” or more per topic category (averaged across items)		Topic category most frequently of top importance per respondent	
	Category	Percentage	Category	Percentage
1	How well the vaccines work	94%	How well the vaccines work	46%
2	Overall COVID-19 impact	92%	Overall COVID-19 impact	37%
3	Vaccine benefits	91%	Vaccine creation	35%
4	Vaccine creation	90%	Long-term vaccine problems	34%
5	Short-term vaccine side effects	89%	Short-term vaccine side effects	33%
6	Long-term vaccine problems	89%	Vaccine benefits	24%

#### Final Content

**Figure 2:**
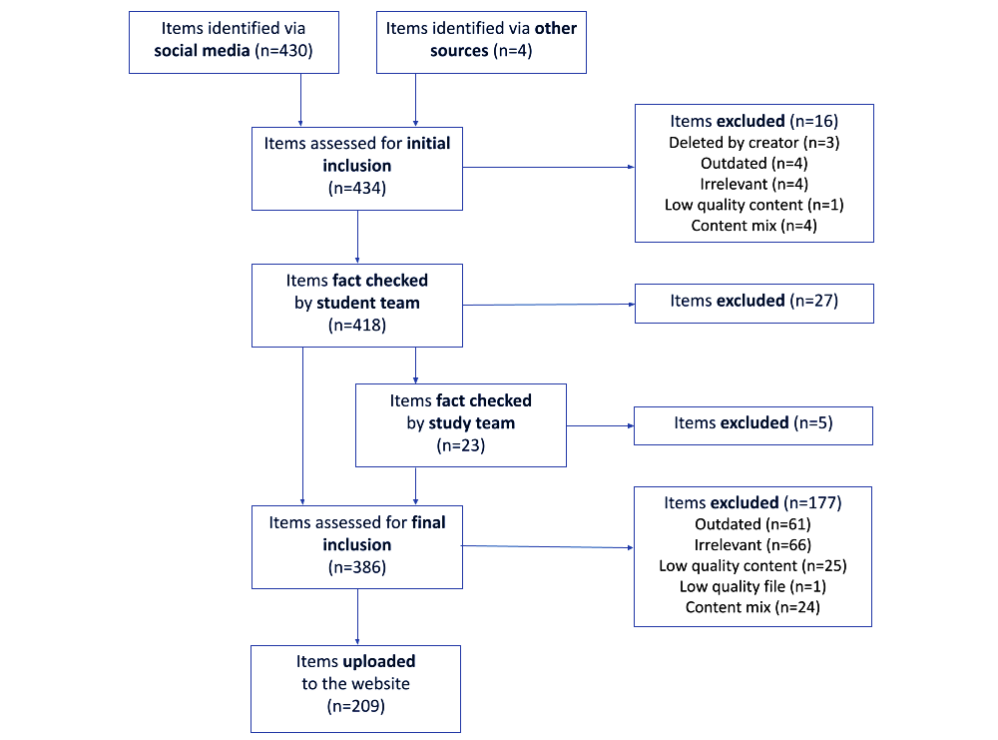
Content identification, fact-checking, and screening flow diagram.

By launch, we had identified 434 content items for the web application, with 209 items ultimately uploaded to the live site (Figure 2). Post characteristics varied (Table 2). Most posts (n=133) were videos, and the remainder were images (n=65) or text-based posts (n=11). The content came largely from Instagram (n=56), TikTok (n=54), and YouTube (n=51), with fewer posts from Facebook, Twitter, and Reddit.

Most posts (n=125) addressed the benefits of getting vaccinated, such as preventing death and illness. About a third (n=75) of the content covered COVID-19 as a disease and the severity of the pandemic. Other posts were about vaccine development (n=39) and the potential risks associated with vaccination (n=35), including side effects and heart problems. Some posts addressed multiple categories. Finally, a minority (n=25) of posts were not specifically related to these topic categories. These posts were largely health care workers’ personal stories, discussions of how vaccine misinformation works in general, or memes from certified nursing assistants or LTCWs.

We worked to reflect the LTCWs’ content mix preferences and the national survey result topics in our final content set, although some preferences competed, including the preference for video but suspicion of sources on TikTok. Given that the concerns about TikTok were related to its perceived untrustworthiness, we worked to rigorously fact-check each post to mitigate this concern.

**Table 2:** Final content characteristics.

Characteristic		Value (N=209), n (%)
**Media type**		
	Video	133 (63.6)
	Image	65 (31.1)
	Text	11 (5.3)
**Media source**		
	Instagram	56 (26.8)
	TikTok	54 (25.8)
	YouTube	51 (24.4)
	Facebook	32 (15.3)
	Twitter	8 (3.8)
	Reddit	6 (2.9)
	Other	2 (1.0)
**Creator role**		
	Medical expert	87 (41.6)
	Journalist	27 (12.9)
	Healthcare organization	22 (10.5)
	Government	20 (9.6)
	Layperson	20 (9.6)
	Long-term care worker or certified nursing assistant	18 (8.6)
	University or education organization	14 (6.7)
	Study team	1 (0.5)
**Topics^a^**		
	Vaccine benefits	125 (59.8)
	About COVID-19	75 (35.9)
	Vaccine creation	39 (18.7)
	Vaccine risks	35 (16.7)
	General	25 (12.0)

^a^Individual content items may address multiple topics.

### Step 2. Platform Development

**Figure 3:**
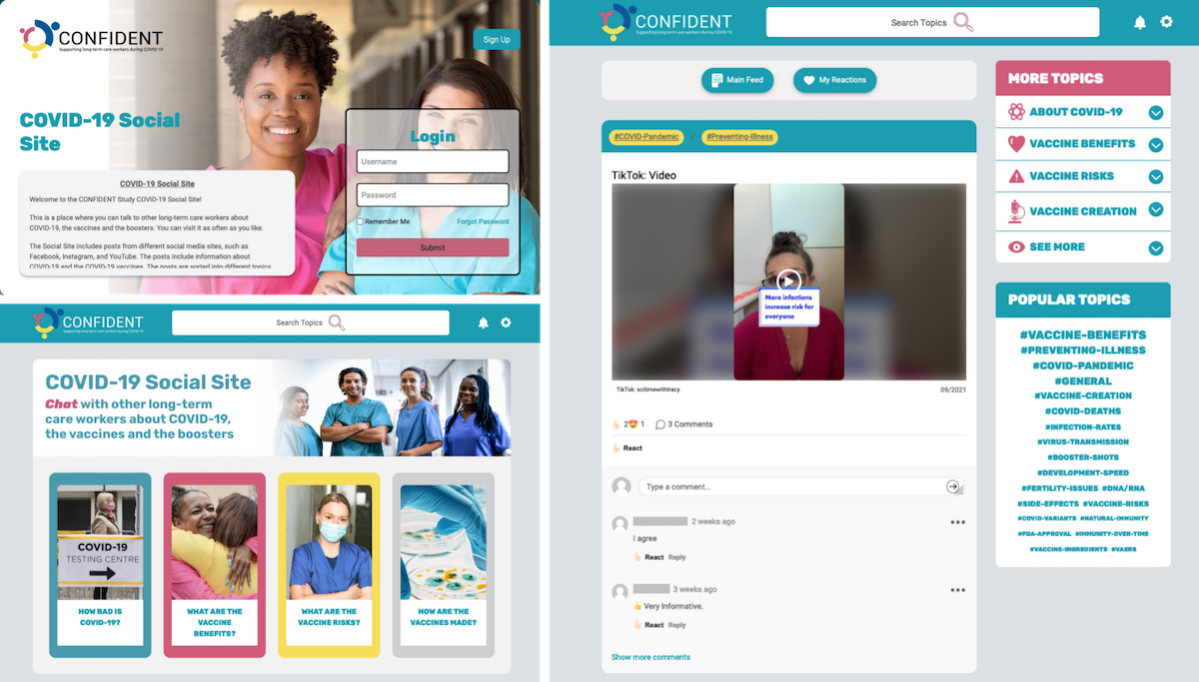
COVID-19 Social Site login and home page.

We created a WordPress-based social media web application called the COVID-19 Social Site (Figure 3). We specifically curated it for LTCWs. It featured an infinite scroll feed with information about each post (title, date, and source). LTCWs could sort posts by topic (level 1) or subtopic (level 2) via an expanding sidebar menu. They could also sort by specific hashtags (level 3).

Users could react to posts with emojis (labeled Like, Love, Haha, Wow, Sad, and Angry). They could also comment on posts and reply or react to other users’ comments. A notification bell alerted users to new activity, and we sent them email updates. We optimized the web application for desktop and mobile use. Interactivity was consistent with other popular social media web applications [15].

Additionally, we tracked user interaction across the social web application using Google Analytics. We informed users of the data collection via cookies with a pop-up dialog box that appeared on their first visit to the site. These web analytics services allowed us to determine which topics or posts received the most views, measure overall user engagement, and troubleshoot navigational or technical issues.

#### LTCW Stakeholder Consultation

LTCW stakeholder consultation participant characteristics are detailed in Step 1. The subthemes that emerged from our interviews with LTCWs related to platform development were that (1) the web application needed to be accessible and easy to use, and that (2) LTCWs should have a safe space to interact with each other, particularly through comments and likes.

#### Accessible and Easy to Use

Although the user habits and levels of digital literacy of the LTCWs and their peers varied, almost all interviewees wanted a clearly laid out web application with simple navigation. We reflected these preferences in the site’s final design, prioritizing ease of use.

#### A Safe Space for LTCWs to Connect

LTCWs wanted the web application to facilitate engagement with other LTCWs via comments. A few participants expressed concerns about the possibility of interpersonal conflict or vaccine-related misinformation but noted that careful moderation by the study team could mitigate this risk. The LTCWs also wanted to interact with posts and comments with likes, emojis, or similar. Multiple interviewees shared that while browsing established social media platforms like Facebook, they were most likely to view and actively engage with posts that had many reactions and comments.

#### UX Testing

We conducted 3 formal UX interviews with LTCWs and various informal UX tests with study team members and stakeholders [26].

We found that users easily recognized the web application as a social media platform and understood the layout. All interviewees agreed that the language and topics featured were valuable. We uncovered various opportunities to decrease user pain points by improving the navigation experience. For instance, we added an instructional video and cues when hovering over the main navigation menus to encourage clicking. Additional details are provided in Multimedia Appendix 9. We did not conduct repeat interviews.

### Step 3. Community Building

#### Community Standards and Moderation

Our final community standards (Multimedia Appendix 10 included guidance about not giving or soliciting medical advice, and avoiding harassment, profanity, hate speech, and spamming. They also encouraged participants to maintain user privacy by not sharing information about others outside the web application.

#### Community Ambassadors

Given the emphasis on a safe space specifically for LTCWs, we decided LTCWs should play a visible role in the web application instead of the research staff, who were present behind the scenes but not visible. Through stakeholder connections, we recruited 3 students as “community ambassadors.” All 3 had long-term care experience and were training to become nursing home administrators.

The ambassadors were special users and community members, empowered by the study team. They used the site freely, commenting and reacting based on their views and positionality as LTCWs.

We asked the community ambassadors to report any comments of concern for individual review by moderators. This approach allowed the site to function as a partially self-moderating community, as regular users could also flag comments for review.

In addition to monitoring the site for community standard violations, we instructed the community ambassadors to encourage participant discussion by actively engaging with other users’ comments. We also asked the community ambassadors to respond to questions and concerns using information relating to COVID-19, the vaccines, and the boosters vetted by the study team (Multimedia Appendix 11).

Additional details on the community standards and the community ambassadors are outlined in Multimedia Appendix 10.

## Discussion

### Principal Findings

We successfully co-developed a novel social media web application featuring curated content specifically tailored to LTCWs with low vaccine confidence. LTCWs are a difficult-to-reach population who we were able to engage through participatory research and user-centered design. Although LTCWs report feeling overlooked professionally, they enthusiastically participated as partners in our project. Testing of the web application in a randomized trial is ongoing.

Through formative semistructured interviews, we found that LTCWs are proud of their work but feel undervalued, particularly in light of the COVID-19 pandemic. They often distrust official sources of information, including the government and popular media. Due to this combination of professional identity and distrust, they welcomed a social media platform specifically customized to them that is trustworthy and easy to understand and use.

To our knowledge, this is the first co-developed web application using curated content from social media to influence attitudes and behaviors about COVID-19 vaccines and boosters. It is also the first web application of its kind specifically for LTCWs, a critically important but understudied population.

### Limitations

We developed the web application in response to the COVID-19 pandemic, a rapidly evolving and urgent public health crisis. Web application development, testing, and launch occurred on an expedited timeline. Under more favorable conditions, we would have conducted more extensive user testing before the trial launch.

A key limitation of our qualitative stakeholder work is that we conducted most of our formative interviews with LTCWs who were already engaged in the project and vaccinated. This sample may have affected the responses. Additionally, LTCWs who agreed to become partners in a vaccine confidence project may be meaningfully different from other LTCWs. While most of our stakeholders were vaccinated, they still had questions and concerns about COVID-19 vaccines and boosters.

Although we designed our national online panel survey to include individuals who were demographically representative of LTCWs, the information preferences of LTCWs may be inherently different from those of the survey participants. Additionally, our content mix poll included a small sample, limiting its representativeness.

Our social media web application, siloed in its own space online, is materially different from most other social media as it exists separately from the rest of the information ecosystem. This separation was necessary to isolate the effects of our intervention and prevent contamination in a larger randomized trial.

### Comparison With Prior Work

Through our user-centered design and participatory research approach, our intervention evolved to reflect other successful interventions in this space more closely, namely a social media web application trialed by Glanz et al that improved vaccine uptake among children of participating parents [10]. This intervention featured vetted information from the study team and ways for participants to interact with each other and the researchers [10].

Other research teams have successfully developed patient-facing communication interventions using participatory research methods [14]. Moderation of the intervention’s content by stakeholders is a participatory research approach we have never implemented before. Participatory research is promising and increasingly popular, and although evidence of efficacy or effectiveness is limited, the evidence base is growing [39-42]. We look forward to contributing to this body of knowledge with our randomized controlled trial results. While meaningful stakeholder engagement in all stages of the intervention development and project may be challenging in a condensed timeline, in the context of a pandemic, the benefits highlighted in the context of our study far outweighed the constraints.

Concerning the qualitative experiences of LTCWs overall, our findings that they feel underappreciated professionally are consistent with the findings in the literature, including research that has emerged during the COVID-19 pandemic [43]. Fisher et al memorably called LTCWs the “forgotten front line” [43]. Other researchers reported that LTCWs felt invisible and unsupported [44]. White et al noticed that although the media portrayed hospital staff as champions, negative media coverage of nursing homes was demoralizing [45]. We believe that our social web application and its co-development process demonstrate that LTCW perspectives truly matter and can shape the content, format, and moderation of a complex intervention designed to improve vaccine confidence among crucial health care workers.

### Next Steps

It is not yet certain that the COVID-19 Social Site will positively influence vaccine confidence or uptake. The results of our randomized trial will be available in 2024.

If successful, our intervention could become a template for other populations with low COVID-19 vaccine confidence or uptake. Additionally, this approach could suit different contexts where changing attitudes or behaviors could be beneficial for public health. Our web application is a light-touch, low-cost intervention that may be relatively easy to replicate and implement by governments, employers, and others. Additionally, the resources associated with launching web applications such as these could be scaled up or down. Without the constraints of a randomized trial, we could also replicate elements of this approach using established social media platforms.

### Conclusions

With user-centered design and participatory research, we developed a novel social media web application featuring curated internet content specifically for LTCWs.

## Appendix

Multimedia Appendix 1 Long-term care worker interview select quotes.

Multimedia Appendix 2 Content identification methods and results.

Multimedia Appendix 3 Long-term care worker interview methods and results.

Multimedia Appendix 4 Long-term care worker interview guide.

Multimedia Appendix 5 Long-term care worker content mix poll questionnaire.

Multimedia Appendix 6 National online panel survey questionnaire.

Multimedia Appendix 7 National online panel survey methods and results.

Multimedia Appendix 8 Fact-checking methods.

Multimedia Appendix 9 User experience testing methods and results.

Multimedia Appendix 10 Community building methods and results.

Multimedia Appendix 11 Abridged COVID-19 fact-checking list.

## Abbreviations

LTCW: long-term care worker
UI: user interface
UX: user experience
