# Supplementary material for: Co-Development of a Web Application (COVID-19 Social Site) for Long-Term Care Workers (“Something for Us”): User-Centered Design and Participatory Research Study
Source: J Med Internet Res. 2022 Sep 22;24(9):e38359. doi: 10.2196/38359 (PMC9506501; doi:10.2196/38359)
Supplement: Multimedia Appendix 1 [file jmir_v24i9e38359_app1.docx]

**Multimedia Appendix 1.** Long-term care worker interview select quotes.

| Themes | | | Select quotes^a^ |
| --- | --- | --- | --- |
| **Major Theme 1. LTCWs^b^ are proud of their work but feel undervalued** | | |  |
|  | Unique vocational  identity as LTCWs | | “I’m also at the transitional journey in somebody’s life and sometimes it’s me that is there at the end. [...] To be invited into a patient’s sacred area, sacred ground and to participate in something so intimate as bathing them, as dressing them and still giving them their dignity, I consider an honor.” [Participant #4]  “This is the only job that I know how to do. This is my passion. This is my career. I can’t imagine myself working at a Walmart or a telephone place. I have to be here.” So, I said, “I’m going to take it or I’m not going to take the vaccine until the last minute. Until they say, [...] “It’s either the vaccine or you lose your job.” I love my job and I love my residents.” [Participant #3] |
|  | Personal connection to and care for residents | | “I love that it’s such a sense of family when we’re there. You know, I see half of them more than I see my family.” [Participant #2]  “If [a LTCW] is the one that introduced it into the facility and [they] could have prevented it somehow, and these 15-ish, 13, 18 people that passed away could have been prevented by [them] getting a vaccination, I’m willing to bet they would have done it.” [Participant #2] |
|  | Work-related COVID-19 distress | | “It was horrible, horrible. I was there, I worked for 30 days straight, 30 days. I went home for six hours just to sleep. That’s how bad it was. [...] It’s horrible to see the suffering that they suffered. Hospitals wouldn’t take them. We couldn’t just send our guys to the hospital. We had to do what we had to do to take care of them. That’s all we could do. [...] We were winging it too because nobody knew.” [Participant #9]  “We were just talking about how COVID took a bunch of people from us [...] When you lose those kinds of people, whether it’s COVID or whether it’s not, it affects the entire community.” [Participant #5] |
| **Major Theme 2. LTCWs have varying levels of trust in COVID-19–related information** | | |  |
|  | Trust in family and friends | | “After a lot of investigation, a lot of reading, a lot of back and forth with my mom, I just decided for the benefit of myself and my family, my husband and my grandchildren that I needed to do this.” [Participant #4]  “It’s people in their community and I think a lot of it was older influences in their family. They may not trust healthcare as much.” [Participant #4] |
|  | Trust in colleagues and LTCW industry leaders | | “He’s our chief medical staff. He’s a really good doctor. He was the first guy that actually got the shot, which is a lot of the reason why I got it in the first place; I watched him do it.” [Participant #5]  “We do receive a lot of emails talking about what’s going on right now about COVID-19 and our information is faster than the others because of my job.” [Participant #6] |
|  | Concerns about vaccine effectiveness, development, and serious reactions | | “Yes, long-term side effects because [...] some of them think even the scientists are not sure what they’re going to get. [...] People question even the structure of the vaccine, the ingredients. They discuss about the RNA vaccine. They ask [...] how they make vaccines previously, the other vaccines, how they made them, how they made this one.” [Participant #9]  “The previous [case] was a resident and he was fully vaccinated. His family wasn’t, they came in to visit, brought it into him, he got sick, and now everybody is like, “Well, why even get the vaccine if it’s not going to help?”” [Participant 7]  “The thing we hear the most here is, “How could they come up with a vaccine within two years of this new virus and have it protect us compared to the stuff that’s been around for generations and we haven’t done hardly anything about it?”” [Participant #7] |
|  | Distrust of institutions, confusion due to contradictory information | | “There are still things about that that I question because I read stuff and then I read something different, and they seem equally as competent and they are conflicting.” [Participant #7]  “African American families do not have a lot of trust and faith in healthcare so that feeling kind of comes down to it.” [Participant #4] |
| **Major Theme 3. LTCWs would welcome a curated resource that is easy to understand and use** | | |  |
|  | **Content** | |  |
|  |  | Carefully curated for LTCWs | “Not a whole lot but something just concise down to two or three sources. [...] As a CNA, we may be lumped into stuff that is directly or really pertain to nurses. [...] Just to make sure that the features are pertaining to your target audience that you’re trying to talk to and I guess just kind of make it as plain of information given as you possibly can.” [Participant #4] |
|  |  | Trustworthy information | “I think if people knew where their information was coming from, it might help to sway what they believe. [...] I think we really need to look at who is funding our research project, or papers, or a study, and also who is publishing the study, as well as who is participating in the study as well. I think the more information people have on the competency of the information being given, the more they are likely to not be drawn into misinformation.” [Participant #2] |
|  |  | Personal stories | “I think maybe including people’s stories that would be willing to share would be something that would be helpful too. Maybe something from personal experiences, not from a doctor, or a lab technician, or a scientist. From real people, what they experienced.” [Participant #2]  “NAHCA always have stories about CNAs, or stories of nursing homes, or a story of residents and I really like reading those kind of stories. I think anything that has to do with my job, or a caregiver, or nursing home is very interesting reading about.” [Participant #3] |
|  | **Topics** | |  |
|  |  | Effectiveness | “It’s honestly one of the bigger arguments I’ve heard now is, “Why should I get the shot if even if I’m vaccinated, I can still get COVID?”” [Participant #5]  “We were just talking about that the other day at work. My friend said that she refused to get the vaccine. She said that she got some family members vaccinated with both vaccines, and they still got COVID and they still died even though they were vaccinated. So then, there comes the big question again, it’s like why do we get the vaccine, if it’s not going to work? We’re still going to get COVID, some people are still going to die. [...]” [Participant #3]  “On the news, they only throw in the people that have died from COVID, but they haven’t really talked about people that are vaccinated that contracted COVID and that they have passed away. [...]The vaccine is not 100% curable[/effective].” [Participant #3] |
|  |  | Development | “I think people hear it’s new, so they immediately associate with “It’s untested.”” [Participant #2]  “It’s like, “Well, I’m going to give you da-da-da-da da-da-da,” and you have no clue what that is [...] and you don’t know what’s in there.” [Participant #4] |
|  |  | Serious reactions | “Mainly, it’s been the, “How is it going to affect me?” Or, “is it going to hurt me?”” [Participant #4]  “A lot of the discomfort comes from the lack of research. What’s going to happen? Am I going to grow some huge lump on this side of my face from getting this vaccine 10 years ago?” [Participant #7] |
|  | **Format** | |  |
|  |  | Plain language | “It's not broken down into what I would call “layman terms.”” [Participant #7]  “Not everyone in that setting understand all these terms. Like if you read some article from CDC, you have like millions of stuff, you – “What is this?” It needs to be something simpler that needs to be quicker to understand in the setting for our target people. [...] We can still put the scientific, but it has to have an aid of simpler and quicker to understand way.” [Participant #1] |
|  |  | Visual information | “I’m definitely a picture person. I think that that’s a lot of this generation too, is if you go to a website and you see all of this text on this page, I’m just going to go right by it, as compared to somewhere that has pictures that you can look at right next to a small paragraph of text.” [Participant #7] |
|  | **Design** | |  |
|  |  | Safe space for LTCWs to connect | “I don’t think it should just be top leaders and that’s it because I’ll tell you, the CNAs don’t listen to them. They don’t listen to administration. [...] They want to hear from people who have been there, seen it, and dealt with it. [...] Yes, and they want their peers.” [Participant #9]  “Threads are always good [...] because there are people that actually want to talk nowadays. Like as crazy as that sounds, people like conversing.” [Participant #5] |
|  |  | Accessible and easy to use | “I find websites the easiest to use when there’s like a tab system at the top – “Click here for stories,” “Click here for bios,” “Click here for case studies,” “Click here for…” and it’s all just laid out right at the top on the main page. So, nothing is hidden. Everything is accessible all in one spot.” [Participant #2]  “I want to be able to find what I want to find within the first minute that I’m on that page rather than having to search for it.” [Participant #7] |

^a^Quotes are edited for length and clarity.

^b^LTCW: long-term care worker.
