# Supplementary material for: Co-Development of a Web Application (COVID-19 Social Site) for Long-Term Care Workers (“Something for Us”): User-Centered Design and Participatory Research Study
Source: J Med Internet Res. 2022 Sep 22;24(9):e38359. doi: 10.2196/38359 (PMC9506501; doi:10.2196/38359)
Supplement: Multimedia Appendix 2 [file jmir_v24i9e38359_app2.docx]

### **Methods**

#### ***Desktop research***

To guide content sourcing for the Social Site, we generated a hierarchy of questions and concerns about COVID-19, the COVID-19 vaccines and the boosters among those with low vaccine confidence. We identified these areas by monitoring peer-reviewed articles, public opinion polls and social media discussions. We then refined this hierarchy with feedback from our LTCW partners.

Specifically, we tracked new developments in the peer-reviewed literature and U.S. attitudinal polling on PubMed (MEDLINE) and the Kaiser Family Foundation’s COVID-19 Vaccine Monitor, respectively.

We also examined select social media communities, including but not limited to the Facebook groups Vaccine Talk and CICADA. Both are large communities dedicated to discussing vaccines. Vaccine Talk invites evidence-based debate from those for and against vaccines, while CICADA responds to vaccine misinformation. Other Facebook groups we monitored included Vaccines Save Lives, I Got the COVID Vaccine! and the COVID Vaccine Discussion Group. By tracking user discussion in these communities and other popular social media platforms, we could better identify emerging concerns.

The process of refining the content categories with our LTCW advisory group ultimately resulted in four topics, which were displayed prominently at the top of the main feed in the web app (Figure S1).

### **Figure S1. COVID-19 Social Site home page content category cards**


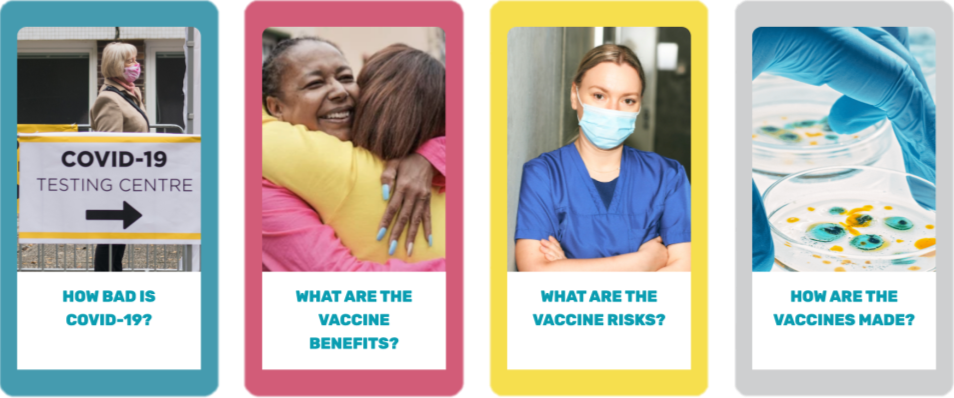


#### ***Search strategy***

Our content team included undergraduate students and graduate researchers interested in public health and social media. After identifying common areas of concern regarding COVID-19 vaccines, we began sourcing the content that would appear on the Social Site. We looked for posts from several social media sites, including Facebook, Instagram, Reddit, TikTok, Twitter and YouTube.

Along with general browsing, we monitored algorithmic feeds, such as TikTok’s “For You” and Instagram’s “Explore” pages. We searched for hashtags related to both broader topics (e.g. #covid, #covid19, #vaccine) and emerging concerns or areas of misinformation (e.g. #myocarditis, #microchips, #vaers). In the case of Reddit, which is organized as a series of user-created discussion boards, we targeted the largest of the relevant ‘subreddits’ (e.g., r/COVID19, r/Coronavirus, r/CoronavirusUS, r/DebateVaccines, r/dataisbeautiful). We also identified posts from healthcare workers, including CNAs. As we scanned for content, we sought the most popular posts based on user engagement metrics, such as likes, shares and comments.

Ongoing content sourcing mirrored the user experience of other social media sites. It also allowed us to adapt content regarding COVID-19 given the dynamic public health environment.

### **Results**

At the time of its launch, we had sourced 434 content items for the web-app. Of this set, 418 passed the initial stage of screening for inclusion. After fact-checking, 386 items progressed to the final screening stage for inclusion based on LTCW content mix criteria. Ultimately, we uploaded 209 items to the live web-app. We continued to source new content throughout the study, updating the feed.

We worked to reflect the LTCWs’ content mix preferences in our final content set, although some preferences competed, including the preference for video but suspicion of sources on TikTok. Given the concerns about TikTok were related to trustworthiness, we worked to rigorously fact-check each post in an attempt to mitigate this concern. We continued to work towards addressing content mix preferences throughout the project.
