# Supplementary material for: Co-Development of a Web Application (COVID-19 Social Site) for Long-Term Care Workers (“Something for Us”): User-Centered Design and Participatory Research Study
Source: J Med Internet Res. 2022 Sep 22;24(9):e38359. doi: 10.2196/38359 (PMC9506501; doi:10.2196/38359)
Supplement: Multimedia Appendix 3 [file jmir_v24i9e38359_app3.docx]

### **Methods**

#### ***Participants***

We purposively recruited interviewees from our LTCW partners across key demographic factors including race and ethnicity, gender, preferred language and employment characteristics such as role, facility type, and location. MC, who has a leadership position at an LTC-related association, approached potential interviewees via online correspondence. MC's outreach resulted in six participants. MH and RL recruited the remaining participants. MH works as a patient advocate, and RL is a health services researcher with experience as an LTC licensed administrator. All of the individuals we approached consented and participated. Before the interviews, we spoke with each participant over Zoom or the phone to ensure comfort and understanding of the study and all interview procedures.

#### ***Procedures***

We developed and piloted a semi-structured interview guide with input from MC and RL. MH and JH conducted all interviews via Zoom and audio recorded the conversations. A colleague of the interviewers was also present during the sessions to provide technical support.

JH is a female quality improvement expert. MH is a female patient advocate with extensive experience in patient engagement, education and web design. MH and JH have training and experience in focus groups and qualitative interviews. They have also both worked in long-term care facilities during the COVID-19 pandemic. MH and JH shared their professional backgrounds and roles in the study with participants at the start of each interview.

We did not carry out repeat interviews. We stored all data in a secure online file sharing platform. The interviewers reviewed transcripts and assessed data saturation periodically. After determining sufficient saturation by the seventh interview, MH and JH conducted two more to confirm, resulting in nine interviews. Participants did not provide comments on the transcripts, but we did iteratively discuss the findings with them.

#### ***Analysis***

We conducted an inductive thematic analysis[[22,23]](https://paperpile.com/c/mLNCUH/aHa5+DI6T). Three female health services researchers coded independently using ATLAS.ti. AS, JP and CHS have a masters in social research methods, a master's in public health, and a Ph.D. in health services research, respectively. The coders processed a sample of all interview data, developing descriptive codes as they progressed. They met as a team to combine their codes into a code hierarchy. Once the codebook was stable, they coded the remainder of the data.

AS, JP and CHS performed thematic analysis collaboratively, along with MAD. Each coder came to the meeting with proposed, independently generated themes and supporting quotations. We shared major and minor themes, engaged in robust discussion and came to a consensus on the themes represented in the dataset.

Once our themes were stable, we revisited the codebook and supporting data to ensure we had not missed any relevant themes or important theme-disconfirming data.

### **Results**

The LTCWs interviewed included five females and four males. Four participants reported they were White, two Asian, two Black, and one biracial. All LTCWs indicated English as their preferred language, except for one who selected Cantonese. Interviews lasted approximately 60 minutes and occurred over Zoom between September 16 and November 5, 2021.

Our codes represented complete thoughts or descriptive statements about the data. Codes fell into categories, including vaccine confidence influences, topics of interest, and web-app functionalities of interest.
