# Supplementary material for: Co-Development of a Web Application (COVID-19 Social Site) for Long-Term Care Workers (“Something for Us”): User-Centered Design and Participatory Research Study
Source: J Med Internet Res. 2022 Sep 22;24(9):e38359. doi: 10.2196/38359 (PMC9506501; doi:10.2196/38359)
Supplement: Multimedia Appendix 4 [file jmir_v24i9e38359_app4.docx]

**Long-Term Care Worker/Stakeholder**

**Intervention Needs Assessment Interviews**

**Interview Guide**

# Notes for interviewers

| **On Redirection**  Here are a few examples on how to redirect the interviewee if they begin to go off topic and away from the content of the interview:   - Acknowledge what the participant has said then redirect the conversation. - Rephrase what the participant said to acknowledge her thoughts then can say “let’s take a step back” and then repeat the question. - Can say thank you for sharing if it feels appropriate.  **On Probing**  All probes are in italics and gray text. See below.   - *This is what the probes look like.*  **Instructions** Instructions for interviewers are in orange text. See below. This is what the text for interviewers looks like. |
| --- |

# Introduction

## **About the study**

I want to tell you how much I appreciate you sharing your time with us. You’ll be helping us learn how to give long-term care workers (LTCWs) information that will help them feel confident about the COVID-19 vaccines.

Specifically, for the CONFIDENT study, we plan to compare two programs to improve confidence in the COVID-19 vaccines. Today, we’ll be talking about building a website with social media posts and creating a webinar specifically for LTCWs.

We’re really interested in your perspective about what would be good to include in these programs, including the topics and also the ways we present the information. We will also ask you about your views on, and experiences with, COVID-19 and the COVID-19 vaccines.

## **Information sheet**

The information sheet we emailed you explained that your participation is voluntary and you can skip any questions that you’d like during the interview. Do you have any questions about the information sheet or concerns about your participation?

*Review any questions they have.*

## **Questions**

If you have any questions during the interview, please just stop me and ask. You can also stop the interview at any time.

###

### **Do you have any questions before we begin?**

*Review any questions they have.*

## **Recording**

### Lastly, we’d like to record this conversation to capture everything you say. We’ll just record the audio, no video, and the recording will only be used by our research team to refer back to your question responses and recommendations.

###

### **Are you comfortable with me recording our conversation?**

*Based on the answer, turn the recorder on. Check to be sure the recorder is on. Proceed with the interview. Take detailed notes if they say no to recording.*

Background

(weather question/small talk)

**Can you start by telling me a little about your role in the [insert relevant work location, such as nursing home, assisted-living facility] and why you got into this work?**

Content

Overall questions and concerns

Now, we want to understand common questions and concerns people have about the COVID-19 vaccines, so we know what to address in our webinar and social media programs.

### **Could you tell me about what influences your opinion about the COVID-19 vaccines? You don’t need to tell me if you’re vaccinated or not unless you want to.**

#### *Did your personal experiences with COVID-19 or the vaccine affect your decision?*

#### *What about your family or friends? (social)*

#### *Your community? (cultural)*

#### *Information from social media? Information from the news media?*

#### *Any particular information?*

#### *Your job*

###

### **What about the people you work with? What do you think influences their opinions about the COVID-19 vaccines?**

#### *Their personal experiences with COVID-19 or the vaccine?*

#### *What about family or friends? (social)*

#### *Community? (cultural)*

#### *Information from social media or news media?*

#### *Any particular information?*

#### *Job (professional identity)*

### **Based on what we’ve talked about so far, what overall questions and concerns do you think are most important?**

I want to share some concerns we have heard from people about getting the vaccine and ask if you think we should address them in our programs.

**Access and process**

**Some people are concerned about where to get the vaccine, how much it costs, and what it’s like to get the shot.**

- (if they have further questions) Some people wonder if the vaccine will hurt. Or if they will have difficulty finding a place to get vaccinated.

**Do you think this is an important topic to address?**

- Why? Or why not?

**What sort of information do you think we should provide in response?**

- Are there any related questions or concerns we should address?

**Information**

**We hear a lot that people have concerns about finding the right COVID-19 vaccine information.**

- (if they have further questions) Some people have trouble finding information or finding information they can understand. Others have trouble finding information in the right language.

### **Do you think this is an important topic to address?**

#### *Why? Or why not?*

###

### **What sort of information do you think we should provide in response?**

#### *Are there any related questions or concerns we should address?*

#### *What do you think about tone? Should it be conversational or scientific?*

##

## **Benefits and efficacy**

### **We’ve also heard that people have questions and concerns about how well the COVID-19 vaccines work. People wonder if they can still get COVID-19 after they’ve been vaccinated and whether they could still get sick or even die.**

###

### **Do you think this is an important topic to address?**

#### *Why? Or why not?*

####

### **What sort of information do you think we should provide in response?**

#### *Are there any related questions or concerns we should address?*

##

## **Side effects and harms**

### **We’ve also heard that people have questions and concerns about side effects, both short-term and long-term.**

#### (if they have further questions) *Some people ask if the vaccine can cause them to get sick with COVID-19. Others are worried that something might happen in the future, like they may not be able to get pregnant.*

### **Do you think this is an important topic to address?**

#### *Why? Or why not?*

####

### **What sort of information do you think we should provide in response?**

#### *Are there any related questions or concerns we should address?*

## **Development process**

### **What about questions and concerns about the COVID-19 vaccine development process?**

### (if they have further questions) *A lot of people wonder how the vaccine was developed and approved so quickly.*

### **Do you think this is an important topic to address?**

#### *Why? Or why not?*

### **What sort of information do you think we should provide in response?**

#### *Are there any related questions or concerns we should address?*

## **COVID-19 pandemic**

### **We’ve also heard that people have concerns and questions about COVID-19 and the virus that causes it.**

#### (if they have further questions) *Sometimes people wonder if COVID-19 is a serious disease or if the pandemic has been exaggerated. Lately, a lot of people are also wondering about variants of the virus.*

### **Do you think this is an important topic to address?**

- *Why? Or why not?*

### **What sort of information do you think we should provide in response?**

- *Are there any related questions or concerns we should address?*

### **Is there anything we haven’t touched on during this part of our conversation that you think we should talk about?**

### **Have you seen any information or resources about COVID-19 or the COVID-19 vaccines that you think we should look at?**

# Functionalities

As you know, we are building two different programs to help people learn more about the COVID-19 vaccines.

## **Social media website overall**

One of these programs is a social media website, where we’ll post some content that has been featured on social media.

### **What features do you think this sort of website should have?**

#### *If you aren’t sure, that’s okay.*

We’ll now walk through some of our decisions in creating the website. We value any insight or feedback that you have.

## **Content sources**

### **We’ll start with website content. We are planning to source content from social media sites including Facebook, Instagram, YouTube, TikTok, LinkedIn, Twitter, and Reddit.**

### **Are there any other social media sources that you trust?**

#### *Why do you trust [this source]?*

#### *Is it important for you to see the source of the content?*

### **Are there specific people online that you look to for health advice?**

#### *What about on televisioon?*

#### *Or newspapers?*

#### *Any other sources of health information or resources we should know about? If so, what?*

### **Are there any social media sources, including those that I listed, that you don’t trust?**

#### *Why don’t you trust this source?*

## **Functionalities**

Now we’ll discuss some of the possible functions of the website.

**How would you like to interact on the website? It’s okay if you can’t think of anything right now.**

- *Which of these ways seems most comfortable to you?*
  - *Comments? Likes?*
  - *Up votes/ down votes*
  - *Forums? Chat boards? Chat rooms?*
  - *Profile with activity information?*
  - *Most active users on pages/groups?*
  - *Location tagging?*

**Are there other ways you’d like to connect with people that we haven’t already discussed?**

**Would you be interested in receiving notifications from the website?**

- *This could be via an Email or text, or on-screen pop-up.*
  - *Could be triggered by:*
  - *Replies to comments*
  - *Admin/moderator notifications*
  - *New posts*
- If not, what are your concerns?
  - Why not?
- How much anonymity would you like to maintain?

### **How should we organize the content on the website?**

#### *By topics of concern?*

#### *By resource?*

#### *By source?*

#### *Hot content?*

- *Virality?*

## **SDM Webinar**

Now we’re going to talk about our webinar.In these Zoom webinars, we will share information about the COVID-19 vaccines and ask participants to talk about concerns and ask questions. We’d like to get your input and feedback about the features and who to include in the webinar.

## **Facilitators**

We are hoping to have trusted leaders facilitate vaccine conversations.

### **Who do YOU think would be the most trusted leaders for the conversation? For example, it might be a LTCW, a medical or vaccine expert, or someone else.**

#### *What about the people you work with? Who would they trust?*

## **Logistics**

Now I’m going to move on to the logistics of the webinar.

### **How long should the webinar last?**

#### *How long could you stay engaged?*

**Do you think we should email information before the webinar? Specifically, we’re wondering about emailing a tool called an Option Grid, which we will use to share information about the COVID-19 vaccines during the webinars.**

#### *If yes:*

#### *Why?*

#### *Do you think we should use it during the webinar to structure the discussion too?*

#### *If no:*

#### *What are your concerns about sending it out ahead of the webinar?*

#### *Do you think we should use it to structure the discussion during the webinar?*

#### *Do you have any other suggestions about a better time to deliver the Option Grid in relation to the webinar?*

### **How much of the webinar do you think should be spent listening versus having a discussion?**

## **Engagement**

**How would you be most comfortable engaging during the webinar and asking questions?**

#### *It’s okay if you can’t think of anything right now.*

#### *Which of these ways seems most comfortable to you?*

#### *Verbal conversation? Raising your hand online? Using the chat function?*

#### *Should we do introductions?*

- Are there other ways you’d like to connect with people that we haven’t already discussed?

**How much anonymity would you like to maintain?**

#### *What are your thoughts on doing introductions?*

#### *What are your thoughts about having your video on? Should it be required, an option, or not allowed?*

# Your thoughts

We’ll close with a few questions about you and the COVID-19 vaccines.

### **I’d like to understand your vaccination status - are you vaccinated or unvaccinated? It’s okay if you’re not comfortable sharing.**

### **Would you be willing to talk with me about your vaccination decision?**

#### *Did your personal experiences with COVID-19 or the vaccine affect your decision?*

#### *What about your family or friends? (social)*

#### *Your community? (cultural)*

#### *Information from social media? Information from the news media?*

#### *Any particular information?*

#### *Your job*

### **What was your reaction to the mandate that nursing home staff must be vaccinated?**

### **Before we close, are there any questions or concerns you’d like to share with me? Anything you’d like to say that we haven’t touched on today?**

### **Thank you for sharing your time with me today. Your insights and recommendations have been incredibly valuable, and we look forward to continuing to partner with you throughout this project.**

###
