# Supplementary material for: Co-Development of a Web Application (COVID-19 Social Site) for Long-Term Care Workers (“Something for Us”): User-Centered Design and Participatory Research Study
Source: J Med Internet Res. 2022 Sep 22;24(9):e38359. doi: 10.2196/38359 (PMC9506501; doi:10.2196/38359)

###
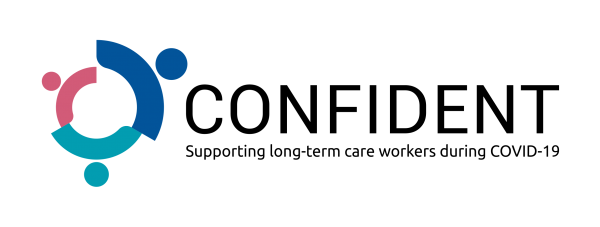


### **Social Media Content Poll**

###

### Start of Block: Introduction

###

### **Thank you!**

The CONFIDENT social media team is busy designing the website intervention, using the feedback the Stakeholder Advisory Group has already provided. Thank you so much for all your help on the CONFIDENT study so far!

###

### **About the social media website intervention**

As a reminder, one of the interventions in the trial will be a social media site featuring curated posts about the vaccines. Visitors to the site will be able to interact with both the content and each other.

**About this poll**

In this poll, we are hoping to get your input about what types of content you would like to see on the social media website. This will help us decide what to put on the website and in what amount. This is a very brief poll and should take you less than 10 minutes. You can skip any questions you are unsure of or would prefer not to answer.

###

### End of Block: Introduction

###

### Start of Block: Default Questions

###

### Q1 How many posts of the following types would you like to see?

|  | None | Some | A lot | No preference |
| --- | --- | --- | --- | --- |
| Videos | ❍ | ❍ | ❍ | ❍ |
| Images | ❍ | ❍ | ❍ | ❍ |
| Infographics (pictures, words, and data together) | ❍ | ❍ | ❍ | ❍ |
| Charts and graphs | ❍ | ❍ | ❍ | ❍ |
| Text | ❍ | ❍ | ❍ | ❍ |
| Other (please specify) | __________________________________________________ | | | |

### Q2 How many posts from the following social media platforms would you like to see?

|  | None | Some | A lot | No preference |
| --- | --- | --- | --- | --- |
| Facebook | ❍ | ❍ | ❍ | ❍ |
| Instagram | ❍ | ❍ | ❍ | ❍ |
| Reddit | ❍ | ❍ | ❍ | ❍ |
| TikTok | ❍ | ❍ | ❍ | ❍ |
| Twitter | ❍ | ❍ | ❍ | ❍ |
| YouTube | ❍ | ❍ | ❍ | ❍ |
| News sites | ❍ | ❍ | ❍ | ❍ |
| Other (please specify) | __________________________________________________ | | | |

### Q3 How many posts from the following types of people would you like to see?

|  | None | Some | A lot | No preference |
| --- | --- | --- | --- | --- |
| CNAs | ❍ | ❍ | ❍ | ❍ |
| Doctors | ❍ | ❍ | ❍ | ❍ |
| Scientists | ❍ | ❍ | ❍ | ❍ |
| Public health experts | ❍ | ❍ | ❍ | ❍ |
| Reporters | ❍ | ❍ | ❍ | ❍ |
| Regular people | ❍ | ❍ | ❍ | ❍ |
| Other (please specify) | __________________________________________________ | | | |

### Q4 How many posts of the following types would you like to see?

|  | None | Some | A lot | No preference |
| --- | --- | --- | --- | --- |
| Scientific data | ❍ | ❍ | ❍ | ❍ |
| News reports | ❍ | ❍ | ❍ | ❍ |
| Personal stories | ❍ | ❍ | ❍ | ❍ |
| Other (please specify) | _________________________________________________ | | | |

### Q5 How many posts of the following types would you like to see?

|  | None | Some | A lot | No preference |
| --- | --- | --- | --- | --- |
| New developments and breaking news | ❍ | ❍ | ❍ | ❍ |
| General information that will stay on the website for a while | ❍ | ❍ | ❍ | ❍ |
| Other (please specify) | _________________________________________________ | | | |

### Q6 How many posts with the following tones would you like to see?

|  | None | Some | A lot | No preference |
| --- | --- | --- | --- | --- |
| Lighthearted | ❍ | ❍ | ❍ | ❍ |
| Serious | ❍ | ❍ | ❍ | ❍ |
| Sad | ❍ | ❍ | ❍ | ❍ |
| Snarky | ❍ | ❍ | ❍ | ❍ |
| Other (please specify) | __________________________________________________ | | | |

###

### Q7 Are you comfortable with posts that contain swearing or curse words?

❍ Yes

❍ No

###

### Q8 Please share any comments or suggestions you may have below.

________________________________________________________________

###

### End of Block: Default Questions

###

##

## **Figure 5. LTCW content mix preferences**

##
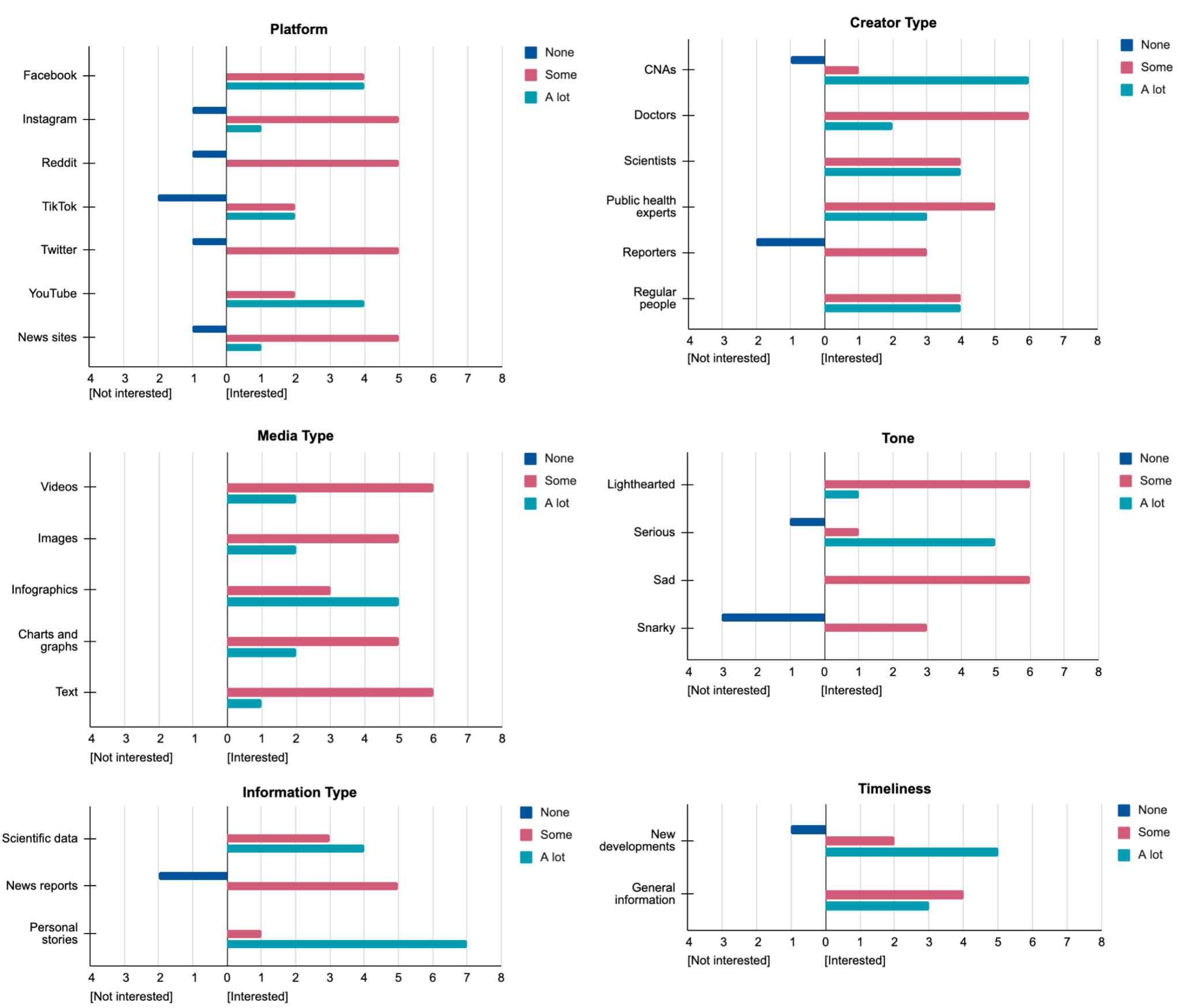

Supplement: Multimedia Appendix 5 [file jmir_v24i9e38359_app5.docx]
