# Supplementary material for: Co-Development of a Web Application (COVID-19 Social Site) for Long-Term Care Workers (“Something for Us”): User-Centered Design and Participatory Research Study
Source: J Med Internet Res. 2022 Sep 22;24(9):e38359. doi: 10.2196/38359 (PMC9506501; doi:10.2196/38359)
Supplement: Multimedia Appendix 6 [file jmir_v24i9e38359_app6.docx]

### **CONFIDENT Planning Survey**

Start of Block: Consent

**What is the goal of this study?** We want to learn about people’s thoughts on the COVID-19 vaccines and vaccines in general. We also want to know what vaccine information is important to them.

**Who is running the study?** Researchers at Dartmouth College led by Professors Glyn Elwyn and Marie-Anne Durand are running the study. We are also working with the Institute for Healthcare Improvement, the National Association of Health Care Assistants, and East Carolina University.

**Who can join the study?** You can join if you are at least 18 years of age and live in the United States. We are looking for a broad range of participants. You will first answer questions about you. Depending on who has answered before you, you may be asked to stop the survey. You won’t need to answer the questions anymore and can close the window.

**What will I have to do?** Joining the study is your choice. You do not have to do it if you do not want to. If you want to do it, we will ask you to answer a 10-15 minute survey. We will ask you questions about yourself and your views on different vaccines. We will also ask you what information is important to know about COVID-19 and the vaccines.

**Will I be paid?** You will get the payment or reward that you agreed upon before you started this survey. No further payments will be given by the study researchers.

**How will you protect my privacy?** You will not need to give any information that would reveal your identity. It will not be possible to identify you when the study results are shared with others or published.

**What are the benefits?** Although you may enjoy answering the questions and sharing your views, you likely will not benefit directly from taking part.

**What are the risks?** We believe the risks are minimal. You might find some of the questions uncomfortable. You may choose to skip any survey questions or stop. In this case, we will use any data you already provided.

**How will you use the survey results?** We will use the results in a larger study. This study is looking at people’s confidence in the COVID-19 vaccines. Results will be shared with others in presentations and publications. Survey responses may also be used for other studies and shared with researchers not part of the group conducting this research project. Survey responses will be stored indefinitely.

**Who should you contact with questions?** Questions about this study should be sent to the principal investigators: Glyn Elwyn (glynelwyn@gmail.com) or Marie-Anne Durand (Marie-Anne.Durand@dartmouth.edu)

Q1 Now that you have read the above information, do you agree to take part in the study?

❍ Yes

❍ No

### If *Yes*, skip to Block: Age

### If *No*, skip to Block: Consent End of Survey

End of Block: Consent

Start of Block: Consent End of Survey

Thank you for your interest in this study.

Based on your response, you are not eligible to participate.

End of Block: Consent End of Survey

Start of Block: Age

Welcome!

First, we’re going to ask you some questions about yourself.

Q2 How old are you?

❍ Less than 18 years

❍ 18-19 years

❍ 20-24 years

❍ 25-29 years

❍ 30-34 years

❍ 35-39 years

❍ 40-44 years

❍ 45-49 years

❍ 50-54 years

❍ 55-59 years

❍ 60-64 years

❍ 65 years and older

If *Less than 18 years*, skip to Block: Age End of Survey

If any other response, skip to Block: State

End of Block: Age

Start of Block: Age End of Survey

Thank you for your interest in this study.

Based on your response, you are not eligible to participate.

End of Block: Age End of Survey

Start of Block: State

Q3 Where do you live?

▼ Alabama [...] Wyoming

❍ I do not reside in the United States

If *I do not reside in the United States*, skip to Block: State End of Survey

If any other response, skip to Block: Race / Ethnicity

End of Block: State

Start of Block: State End of Survey

Thank you for your interest in this study.

Based on your response, you are not eligible to participate.

End of Block: State End of Survey

Start of Block: Race / Ethnicity

Q4 What is your race/ethnicity? Select all that apply.

❍ White

❍ Black or African American

❍ American Indian or Alaska Native

❍ Asian

❍ Native Hawaiian or Other Pacific Islander

❍ Hispanic, Latino/a, or Spanish origin

❍ Some other race(s) or ethnicity(ies). Please specify: _____________

End of Block: Race / Ethnicity

Start of Block: Education

Q5 What is the highest degree or level of school you have completed?

❍ 12th grade or less (no high school diploma)

❍ High school graduate (regular high school diploma, GED, or alternative credential)

❍ Some college credit

❍ Associate’s degree (2-year degree)

❍ Bachelor’s degree (4-year degree)

❍ Graduate degree (Master’s degree, Professional degree, Doctorate)

❍ Other. Please Specify: _____________

End of Block: Education

Start of Block: Gender

Q6 What is your gender?

❍ Female

❍ Male

❍ Non-binary

❍ Prefer to self-describe: _____________

End of Block: Gender

Start of Block: Other Demographics

Q7 Which of the following types of health insurance coverage do you currently have? Select all that apply.

❍ Medicare (a government plan you can enroll in when you turn 65 or have certain health conditions)

❍ Medicaid (a government plan you can quality for based on your income)

❍ Other state-sponsored plan

❍ Private insurance (purchased yourself or provided by your employer)

❍ Indian Health Service

❍ TRICARE or other military health plan

❍ Other. Please specify: _____________

❍ I do not have health insurance

❍ I do not know

Q8 Have you received at least one COVID-19 vaccine shot?

❍ Yes

❍ No

End of Block: Other Demographics

*[ADDITIONAL SURVEY QUESTIONS OMITTED HERE*

*AS THEY ARE NOT DISCUSSED IN THIS MANUSCRIPT]*

Start of Block: Information Intro

Q24 Lastly, we’re going to ask you some questions about how you feel about different types of COVID-19 vaccine information.

End of Block: Information Intro

Start of Block: COVID-19 Vaccine Benefits

Q25 Below is a list of topics about the benefits of the COVID-19 vaccines.

How important is it for you to know about…

|  | Not at all important | A little important | Somewhat important | Very important |
| --- | --- | --- | --- | --- |
| ...your ability to visit public places? | ❍ | ❍ | ❍ | ❍ |
| ...if you can have fewer COVID-19 tests? | ❍ | ❍ | ❍ | ❍ |
| ...your chance of giving COVID-19 to other people? | ❍ | ❍ | ❍ | ❍ |
| ...your ability to visit your friends and family? | ❍ | ❍ | ❍ | ❍ |
| ...your ability to protect yourself from illness caused by COVID-19? | ❍ | ❍ | ❍ | ❍ |
| ...your ability to return to normal pre-pandemic life? | ❍ | ❍ | ❍ | ❍ |
| ...your ability to travel? | ❍ | ❍ | ❍ | ❍ |
| ...your ability to keep your job? | ❍ | ❍ | ❍ | ❍ |
| ...your need to wear a mask less often? | ❍ | ❍ | ❍ | ❍ |
| ...your ability to keep your community healthy, including immunocompromised friends and neighbors with other conditions? | ❍ | ❍ | ❍ | ❍ |

Q26 Are there any other things that would be important for you to know about the benefits of the COVID-19 vaccines?

________________________________________________________________

End of Block: COVID-19 Vaccine Benefits

Start of Block: COVID-19 Information: How Well Vaccines Work

Q27 Below is a list of topics about how well the COVID-19 vaccines work.

How important is it for you to know about…

|  | Not at all important | A little important | Somewhat important | Very important |
| --- | --- | --- | --- | --- |
| ...the protection from the vaccines compared to the protection from having COVID-19? | ❍ | ❍ | ❍ | ❍ |
| ...lessening the spread of COVID-19? | ❍ | ❍ | ❍ | ❍ |
| ...lessening serious illness and death from COVID-19? | ❍ | ❍ | ❍ | ❍ |

Q28 Are there any other things that would be important for you to know about how well COVID-19 vaccines work?

________________________________________________________________

End of Block: COVID-19 Information: How Well Vaccines Work

Start of Block: COVID-19 Information: Side Effects

Q29 Below is a list of topics about the short-term side effects or other possible problems of the COVID-19 vaccines.

How important is it for you to know about…

|  | Not at all important | A little important | Somewhat important | Very important |
| --- | --- | --- | --- | --- |
| ...how often heart problems (e.g., myocarditis) happen? | ❍ | ❍ | ❍ | ❍ |
| ...how often blood clots happen? | ❍ | ❍ | ❍ | ❍ |
| ...how often people have arm pain? | ❍ | ❍ | ❍ | ❍ |
| ...how often people have fevers? | ❍ | ❍ | ❍ | ❍ |
| ...how often people feel tired? | ❍ | ❍ | ❍ | ❍ |
| ...how often people get muscle aches? | ❍ | ❍ | ❍ | ❍ |

Q30 Below is a list of topics about the long-term problems with the COVID-19 vaccines some people are concerned about.

How important is it for you to know about…

|  | Not at all important | A little important | Somewhat important | Very important |
| --- | --- | --- | --- | --- |
| ...if it will lessen your ability to have children? | ❍ | ❍ | ❍ | ❍ |
| ...if there may be unknown long lasting problems? | ❍ | ❍ | ❍ | ❍ |
| ...if you can catch COVID-19 from the vaccines? | ❍ | ❍ | ❍ | ❍ |

Q31 Are there any other things that would be important for you to know about the short-term side effects or long-term problems with the COVID-19 vaccines some people are concerned about?

________________________________________________________________

End of Block: COVID-19 Information: Side Effects

Start of Block: COVID-19 Information: Vaccine Creation

Q32 Below is a list of topics about making the COVID-19 vaccines.

How important is it for you to know…

|  | Not at all important | A little important | Somewhat important | Very important |
| --- | --- | --- | --- | --- |
| ...how fast the vaccines were made? | ❍ | ❍ | ❍ | ❍ |
| ...about the people who made the vaccines? | ❍ | ❍ | ❍ | ❍ |
| ...the ingredients in the vaccines? | ❍ | ❍ | ❍ | ❍ |
| ...the process used to make the vaccines? | ❍ | ❍ | ❍ | ❍ |
| ...the process of vaccine approval? | ❍ | ❍ | ❍ | ❍ |
| ...the history of mRNA vaccine creation? (e.g., Pfizer and Moderna vaccines) | ❍ | ❍ | ❍ | ❍ |

Q33 Are there any other things that would be important for you to know about the creation of the COVID-19 vaccines?

________________________________________________________________

End of Block: COVID-19 Information: Vaccine Creation

Start of Block: COVID-19 Information: Pandemic

Q34 Below is a list of topics about the impact of the COVID-19 pandemic.

How important is it for you to know about…

|  | Not at all important | A little important | Somewhat important | Very important |
| --- | --- | --- | --- | --- |
| ...how bad COVID-19 is for you? | ❍ | ❍ | ❍ | ❍ |
| ...the risks of the vaccine compared to the risks of the COVID-19 illness? | ❍ | ❍ | ❍ | ❍ |
| ...the seriousness of COVID-19 for others? | ❍ | ❍ | ❍ | ❍ |
| ...the impact of COVID-19 variants (such as Delta) on how well vaccines work? | ❍ | ❍ | ❍ | ❍ |
| ...the need for booster shots? | ❍ | ❍ | ❍ | ❍ |

Q35 Are there any other things that would be important for you to know about the COVID-19 pandemic?

________________________________________________________________

End of Block: COVID-19 Information: Pandemic

Start of Block: End of Survey

Thank you for taking part in this study.

Click 'NEXT' to submit your responses.

If you have any questions about this study, please contact the principal investigators: Glyn Elwyn (glynelwyn@gmail.com) or Marie-Anne Durand (Marie-Anne.Durand@dartmouth.edu)

End of Block: End of Survey
