# Supplementary material for: Co-Development of a Web Application (COVID-19 Social Site) for Long-Term Care Workers (“Something for Us”): User-Centered Design and Participatory Research Study
Source: J Med Internet Res. 2022 Sep 22;24(9):e38359. doi: 10.2196/38359 (PMC9506501; doi:10.2196/38359)

### **Methods**

#### ***Participants***

Dartmouth’s Committee for the Protection of Human Subjects (CPHS) approved the study (STUDY00032340). In this cross-sectional survey study, we sampled the U.S. adult population. We sampled so that our population would be demographically similar to the population of LTCW workers on characteristics including age, sex, race and ethnicity, and educational attainment [25].

We chose to sample this way because we did not want to impede on our eligible population of LTCWs for the planned randomized-controlled trial, an already difficult-to-recruit group. Additionally, given the dynamic COVID-19 landscape, we wanted to quickly get baseline insights before launching our broader trial.

#### ***Survey development***

We drafted a survey and piloted it in Qualtrics with our colleagues. Survey developers from Qualtrics reviewed our survey to assist with specific survey flow, functionality, and security settings.

#### ***Survey elements***

We assessed demographics with adapted versions of standard and other questions, including those about age, state of residence [27], race and ethnicity [28], educational attainment [28], gender [29,30], type of health insurance [28,31], and COVID-19 vaccination status. We also asked about participants’ COVID-19 vaccine booster status and intentions, COVID-19 vaccine views and their views about vaccines in general. These questions were included for separate lines of work related to the broader randomized trial and are thus not reported on in this manuscript. Lastly, we assessed participants’ information preferences about COVID-19 and the COVID-19 vaccines across a total of 33 information items [32]. We asked them to evaluate the importance of information across six distinct categories: short and long-term vaccine side effects, vaccine benefits, vaccine development, how well the vaccines work, and the impact of the pandemic overall. These questions were responded to a 4-option Likert-type scale ranging from 1 “not at all important” to 4 “very important”. For each category, participants were also asked to report (via open text) if there were any other things that would be important for them to know.

#### ***Procedures***

##### **Recruitment and consent**

We recruited participants via Qualtrics (www.qualtrics.com, Seattle, WA). Qualtrics is an online survey service. Qualtrics leveraged an Internet panel company which distributed our survey announcement via targeted email invitations, online portals, offerwalls, SMS, or in-app messaging. Those invited to participate/exposed to the survey announcement could have been existing panel members or they may have received an offer to join the panel at that time. Qualtrics also implemented pre-screening questions in order to help them target the desired population for our study. The pre-screening questions assessed for age, gender, race, ethnicity, education, state of residence, and an attention check question. Those who passed pre-screening proceeded to the study survey. Participants consented to the study via an online information sheet.

##### **Incentives**

The panels Qualtrics employs for survey studies offer different incentives, which participants agree to before they start the surveys. These include, but are not limited to, loyalty reward points, gift cards and cash payments.

##### **Survey completion**

Participants entered responses directly into the Qualtrics survey platform. They completed up to 92 questions, including one consent question, six demographic questions, 47 questions relating to views about the COVID-19 vaccines and vaccines in general for the broader randomized controlled trial, and 38 content preference questions. The survey spanned up to 35 screens, though based on their response to the vaccine uptake questions, participants could see as few as 29 screens to complete the survey. We did not use adaptive questioning. Across the whole survey, pages contained anywhere from one to 18 questions.

Several questions included non-response options (e.g. I don’t know, I don’t understand, Not sure). We forced responses on screening and demographic quota questions, but other questions were optional. We did remind participants if they left questions blank. We allowed participants to advance and go back in the survey with a back button, where possible. This was not possible once participants had advanced past most screening and demographic questions or started randomized question blocks. Participants could go back within the randomized blocks, they just could not go back from the randomized block to a previous section of the survey. We randomized the order of some blocks of questions, as well as the order of questions within a block, including (but not limited to) the content preference questions.

Qualtrics and/or the panel services incentivize participants who are considered “good completes”. This includes anyone who completes the survey who is also not considered a “speeder,” which is someone who completes a survey in less than a minimum allocated time. We set the minimum completion time for our survey at 5 minutes, based on pre-survey testing by members of the study team. People are also screened out of a survey and not considered good completes if they: i) exceed a demographic quota that has been filled while they are completing a survey, and ii) do not pass an automated security check which assesses for duplicates (people trying to take the survey more than once) and bots [26]. Qualtrics uses Google’s invisible reCaptcha technology to detect whether a respondent is likely a bot. They also use a function called Relevant ID to detect duplicates and bots. Relevant ID uses respondent metadata based on their browser, operating system and location. Scores are calculated for both the reCaptcha and Relevant ID fields and are used to determine if a respondent will be screened out of a survey or not.

##### **Data protection**

The survey data collected were anonymous. We did not collect or store participants' IP addresses. All raw data stored in Qualtrics could only be accessed by authorized users. Study team members accessed Qualtrics data via a secure login with two-factor authentication. Transport Layer Security (TLS) encryption is used by Qualtrics for all data that is transmitted.

#### ***Analysis***

We explored the content item mean scores and proportion of people reporting each item as “Not at all important”, “A little important”, “Somewhat important”, or “Very important”, when making decisions about which content would be suitable for the social web-app. Our approach was more focused on excluding information content that was not considered important versus deciding what content to include.

We also evaluated the percentage of respondents indicating each item in a category was at least “a little important,” and averaged the percentages for all items within a category to determine the relative importance of each category. We also looked at which categories were most frequently of top importance to respondents. If a respondent scored a greater proportion of items in one category as important (defined as a score of at least ‘A little important’), this category was then identified as the respondent’s most important category (ties were allowed). Across respondents, the frequency of each category being the most important category was determined to identify the areas of greatest interest.

### **Results**

Reasons for not completing the survey included screen outs due to a) ineligibility (n=7), b) failed security check (n=40), or c) full demographic quotas (n=305). In addition, 40 people did not complete the survey due to unknown reasons. Of those who completed the survey, 151 were automatically excluded based on suspiciously fast survey completion time. The final study sample included 592 participants who completed the surveys in December 2021 (Figure 6). Our survey company was not able to reliably provide accurate response rates.

## **Figure 6. National online panel survey participant flow diagram**


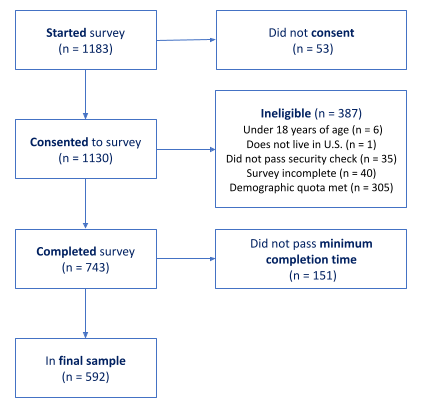


Of those who completed the survey, the majority were female (78.3%) and under the age of 50 (65.9%). Participants did not differ significantly from the national U.S LTCW population on gender, age, race and ethnicity, or educational attainment (Table 4)[25]. About half (49.8%) of participants resided in the South, with the remaining 50.2% split fairly evenly across the Midwest (19.6%), Northeast (15.0%), and the West (15.5%). Sixty four percent (63.9%) of the sample reported receiving at least one COVID-19 vaccine shot.

## **Table 4. National online panel survey participant characteristics**

| **Characteristic** | **N (%)** | **U.S. National^d^ (%)** | **Population Comparison^e^** |
| --- | --- | --- | --- |
| **Gender^a^** | | | |
| Female | 463 (78.3) | (82) | *P*=.15 |
| Male | 118 (20.0) | (18) |  |
| Non-binary or Prefer to self-describe | 10 (1.7) | - |  |
| *Missing* | *1* | - |  |
| **Age** | | | |
| less than 50 years | 390 (65.9) | (62) | *P*=.06 |
| 50 to 64 years | 151 (25.5) | (30) |  |
| 65 years and older | 51 (8.6) | (8) |  |
| **Race/ethnicity^b^** | | | |
| White only | 326 (55.3) | (52) | *P*=.28 |
| Black only | 139 (23.6) | (26) |  |
| Hispanic^c^ | 71 (12.0) | (13) |  |
| Other | 54 (9.2) | (8) |  |
| *Missing* | *2* | - |  |
| **Education level** | | | |
| Less than high school | 43 (7.3) | (10) | *P*=.09 |
| High school graduate | 184 (31.1) | (29) |  |
| Some college | 226 (38.2) | (39) |  |
| Bachelor’s degree or more | 136 (23.0) | (21) |  |
| Other | 3 (0.5) | - |  |
| **Place of residence** |  |  |  |
| Midwest | 116 (19.6) | - |  |
| Northeast | 89 (15.0) | - |  |
| South | 295 (49.8) | - |  |
| West | 92 (15.5) | - |  |
| **Health insurance coverage^b^** | | | |
| Public only | 330 (55.7) | - |  |
| Private only | 164 (27.7) | - |  |
| Other/Don’t know | 41 (6.9) | - |  |
| Uninsured | 57 (9.6) | - |  |
| **COVID-19 vaccination status** | | | |
| Received at least one COVID-19 shot | 378 (63.9) | - |  |
| Did not receive any COVID-19 shots | 214 (36.1) | - |  |
| ^a^We chose to assess gender which differs from national data which was based on an assessment of sex; ^b^Multiple selections were allowed; ^c^Hispanic for the study sample includes people who selected Hispanic, Latino and/or Spanish origin with or without a race selected. Categorization of Hispanic in national data was unclear. ^d^National data were derived from a Kaiser Family Foundation analysis of the American Community Survey, 2018][25]. ^e^For gender and education, study sample percentages used for population comparisons differed slightly to that presented in this table as national LTCW data were not available for some categories. | | | |

The COVID-19 vaccine topics of interest varied among participants (Figure 7).

### **Figure 7. National online panel survey participant topic preferences**


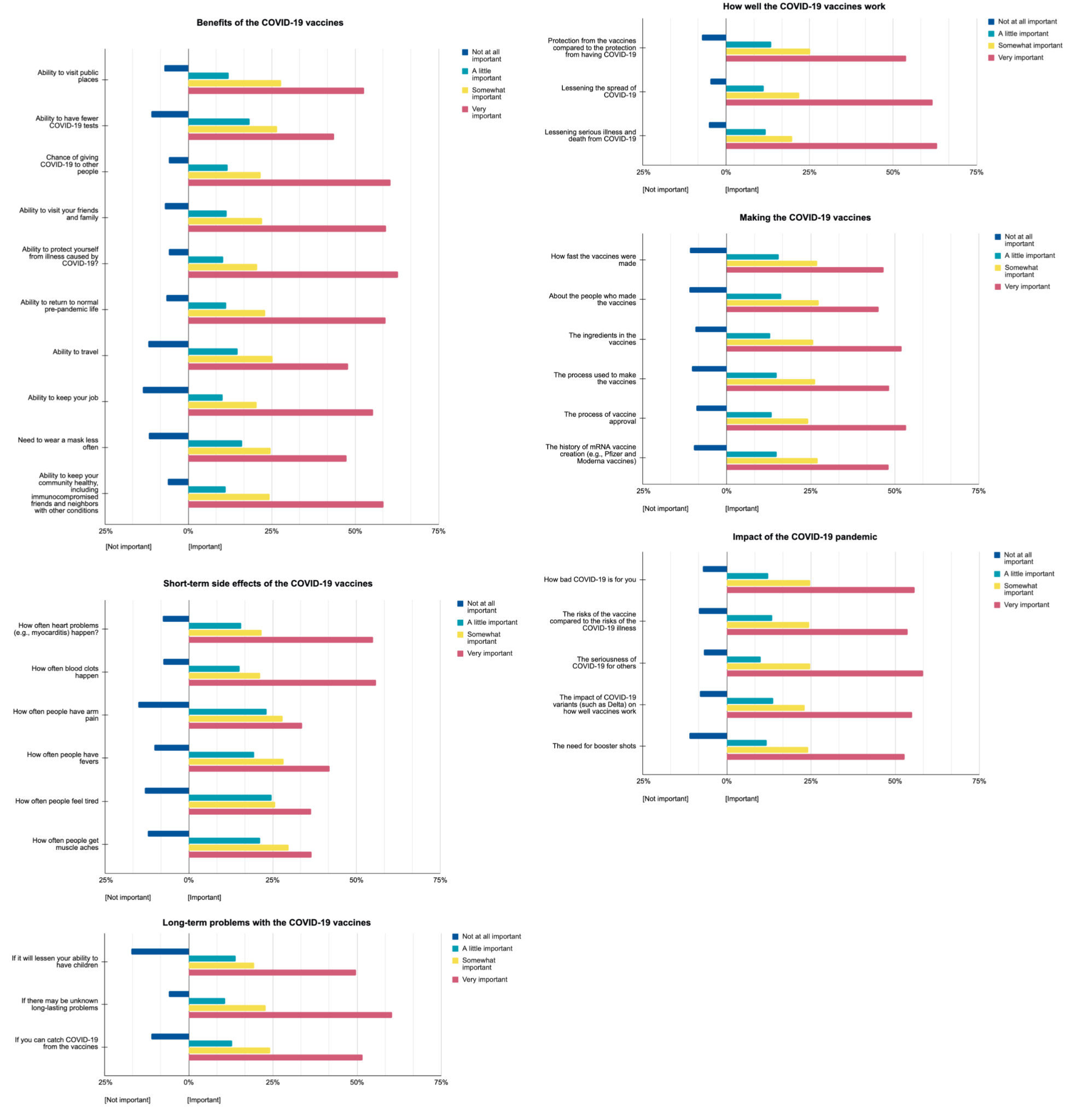

Supplement: Multimedia Appendix 7 [file jmir_v24i9e38359_app7.docx]
