# Supplementary material for: Co-Development of a Web Application (COVID-19 Social Site) for Long-Term Care Workers (“Something for Us”): User-Centered Design and Participatory Research Study
Source: J Med Internet Res. 2022 Sep 22;24(9):e38359. doi: 10.2196/38359 (PMC9506501; doi:10.2196/38359)
Supplement: Multimedia Appendix 8 [file jmir_v24i9e38359_app8.docx]

### **Methods**

We tracked and processed all content using a shared spreadsheet. The content team identified social media posts of interest and logged them along with basic details (e.g. date posted, platform of origin, creator role, user engagement metrics, etc.). We used a category-based system with hashtags.

We designed a fact-checking process in consultation with our broader advisory group, including LTCW partners, medical experts and other stakeholders. We developed a list of trusted up-to-date sources of information on the COVID-19 pandemic and its vaccines, which we then used to check the accuracy of each post. This included the websites of government agencies like the Centers for Disease Control & Prevention, the World Health Organization, and the Los Angeles County Department of Public Health. We also included medical institutions like Yale Medicine, non-profit organizations such as the Kaiser Family Foundation, and newspapers like *The New York Times.* Additional sources were continuously added to the list throughout the fact-checking process.

The content team then used these sources to fact-check each item. If they were unable to determine a post’s accuracy, the item was escalated to study team members CHS and AS for a second round of fact-checking. CHS holds a Ph.D. in health services research, and AS has an MSc in social research methods. If needed, CHS and AS escalated to TH, a geriatrician with expertise in COVID-19 vaccine communication. Either CHS or AS reviewed each post to confirm appropriateness in light of LTCW content mix preferences.

Throughout the fact-checking process, the content and study teams compiled relevant facts into a shared document to expedite further fact-checking. We sorted facts by topic and noted the source and date published. We then transferred the facts to an abridged document for quick reference (Multimedia Appendix 11). This aided in our ongoing monitoring of content for accuracy as the guidelines and research surrounding the pandemic and the COVID-19 vaccines evolved over the course of intervention development.
