# Supplementary material for: Co-Development of a Web Application (COVID-19 Social Site) for Long-Term Care Workers (“Something for Us”): User-Centered Design and Participatory Research Study
Source: J Med Internet Res. 2022 Sep 22;24(9):e38359. doi: 10.2196/38359 (PMC9506501; doi:10.2196/38359)
Supplement: Multimedia Appendix 9 [file jmir_v24i9e38359_app9.docx]

### **Methods**

#### ***Participants***

We purposively recruited participants for user experience testing, with a particular emphasis on diversity in interviewee age. MC, who has a leadership position at an association for LTCWs, approached members of said association as potential participants via online correspondence. The remaining interviewee was a LTCW administration student of RL, a health services researcher.

#### ***Procedures***

We interviewed and observed participants’ behavior on the site via Zoom and its screen sharing feature. We recorded all the testing sessions. CM, a female user experience designer with experience in web design and user research, conducted all interviews.

We used the think-aloud technique as well as standard UX testing procedures, including semi-structured interview questions and observations in real-time as individuals navigated the site[[33,34]](https://paperpile.com/c/mLNCUH/VEBz+6QHL). The interviewer asked participants to perform various tasks on the test site to identify navigational challenges, comprehension issues and visitation patterns. We also asked about preferred features and content areas.

The questions were not pilot tested, as we used standard prompts common in UX interviewing[[35,36]](https://paperpile.com/c/mLNCUH/3fbx+em6P). During and after each session, the interviewer noted participants’ actions on the site, as well as direct quotes. No one else was present during the interviews. We did not assess data saturation, and participants did not provide additional feedback after UX testing was completed.

#### ***Analysis***

We synthesized our findings using an affinity map. Affinity mapping is a well-established technique in UX research used to identify features or patterns that need improvement to increase users’ ease of accessing and interacting with interactive products like webpages[[37]](https://paperpile.com/c/mLNCUH/yEVQ). CM and JH, a lead UX designer, developed the affinity map collaboratively. JH first created a map template, then CM noted users’ behaviors on the map. They then assessed the value of each category and analyzed the map to determine what changes needed to be made.

### **Results**

We conducted three formal UX interviews and a variety of informal UX tests with team members and stakeholders, given the accelerated timeline[[26]](https://paperpile.com/c/mLNCUH/1M4j). All interviewees were female, chose English as their preferred language, and were either White (2) or Black (1). The interviews lasted approximately 35-45 minutes and were conducted over Zoom between January 12 and January 17, 2022. Two individuals declined our invitation due to time constraints, while two others did not respond.

We found users understood the overall layout and easily recognized it as a social media website. They unanimously agreed that the language used and topics featured were valuable and fostered learning about COVID-19 and its vaccines. They felt that there was no moral judgment or moral persuasion implied in the language.

Users also unanimously agreed upon the importance of comment moderation. The users brought up the value of moderation without a prompt from the interviewer, a very strong signal that moderation needed to be well-considered in the design and development of the site. Community guidelines are clearly communicated with all users on the signup and login pages, which outline the rules for using the site. They also state that administrators will be removing any comments that violate these guidelines.

We uncovered opportunities for improving engagement by directly observing users’ interactions with the site as they clicked through the home and topic pages. For instance, users initially hovered over the content category cards (Figure 2) but did not click on them. We addressed this by adding a message to ‘explore topics’ that appears when users hover over the cards, signaling that clicking reveals more information. Users also did not expand the 'more topics' accordion menu to access additional topics, which prompted us to change the color of the directional expansion arrows to ensure they stood out.

To further improve overall clarity of the site’s features, we also developed an instructional tutorial video to play upon users’ first login. It features a screen recording introducing the website and a voiceover explaining how to navigate it, as well as where the social media content originated.
