# Supplementary material for: Co-Development of a Web Application (COVID-19 Social Site) for Long-Term Care Workers (“Something for Us”): User-Centered Design and Participatory Research Study
Source: J Med Internet Res. 2022 Sep 22;24(9):e38359. doi: 10.2196/38359 (PMC9506501; doi:10.2196/38359)
Supplement: Multimedia Appendix 10 [file jmir_v24i9e38359_app10.docx]

### **Methods**

To guide the effort to build a sense of community on the COVID-19 Social Site, we reviewed the community standards of popular Facebook groups focusing on COVID-19 vaccine discussion, including Vaccine Talk: An Evidence Based Discussion Forum and CICADA (Community Immunity Champions and Defenders Association). The former was of particular interest as it explicitly allows posts both in favor of and against vaccines. This is consistent with our intention for the Social Site to be a space where those hesitant about the COVID-19 vaccines would feel comfortable sharing their concerns and asking questions. We referenced the default rules for Facebook groups in general as well.

We then used these to draft an abbreviated set of similar community standards for the Social Site. We also added research-relevant privacy language and used plain language principles to refine the wording[[21]](https://paperpile.com/c/mLNCUH/5a3P).

As we heard from our LTCW partners that they wanted to know that study-affiliated individuals were monitoring and vetting the site’s content and comments, we also created a community ambassador program as a light-touch moderation approach. Our LTCW partners provided iterative feedback on both elements.

### **Results**

Several LTCWs mentioned wanting careful study team moderation during UX testing. For example, one LTCW interviewee expressed concern about an unmoderated environment, stating that “I like a forum where you can get other people’s opinion, but that can also be dangerous because [...] people offer information [...] that is not accurate, so you also have to take it with a grain of salt” (participant 2). During UX testing, users also unanimously agreed on the importance of comment moderation on the Social Site during UX testing, without prompting.

We clearly posted the community standards on both the signup and login pages (Textbox S1). Upon initially creating an account, users were also required to tick a checkbox stating that they agreed to follow the standards. We modeled this after the signup pages of Facebook groups and other popular discussion sites.

### **Textbox S1. Community standards and welcome message**

| Welcome to the CONFIDENT Study COVID-19 Social Site!  This is a place where you can talk to other long-term care workers about COVID-19, the vaccines and the boosters. You can visit it as often as you like.  The Social Site includes posts from different social media sites, such as Facebook, Instagram, and YouTube. The posts include information about COVID-19 and the COVID-19 vaccines. The posts are sorted into different topics, so you can choose to look at what matters most to you.  To use it, you will need to share your email address, and create a username and password. Other users will see your username. It’s up to you if you use your real name.  We will use your email address to send you website notifications, unless you opt out. We will record your activity on the website. We will also link this activity to what you tell us in surveys. We will never tell anyone who you are when sharing study results.  Here are things to avoid so that everyone feels welcome and comfortable. We call these our Social Site Community Standards.   - No medical advice. Please do not ask for or offer medical advice. - No harassment. It’s ok to disagree, but please do not personally attack one another. - No profanity or hate speech. Please do not use profanity and racial/ethnic/religious/other slurs. - No spam. Please do not make too many off-topic posts. Avoid asking so many questions that it is hard for others to answer fast enough.   The study team (admins) can change these rules. They can also limit your activity on the Social Site if you do not follow the Community Standards. |
| --- |

If a comment violated any of the community standards, moderators CHS and AS would manually remove it. This triggered an automated email informing the user that their comment had been removed. If a user repeatedly violated community standards, we could mute their accounts. This would also result in an email stating the user was now blocked from making additional comments.

We also recruited three students with LTCW experience to act as community ambassadors. They ambassadors were instructed to interact with the users on the site by commenting and reacting based on their own views and positionality (Textbox S2).

### **Textbox S2. Community ambassador responsibilities and select quotes**

| 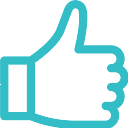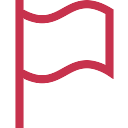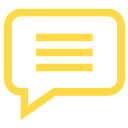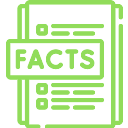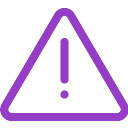 | - Be friendly and welcoming: react and reply to content and participant comments. - Encourage participant discussion: ask participants to engage by making comments and sharing views. - Share factual information: ask permission to share more information when participants have questions. Share information in your own words from our study fact sheet. - Report violations: flag comments that violate community standards via the report function in WordPress. - Flag any other issues: contact the study team via email. | “This was very comforting and validating. I remember being hesitant about the speed of production for the COVID-19 vaccine as well. I found much comfort in discovering that there were years of research on SARS and mRNA technology.”  “This was very helpful, emphasizing the good that comes from the vaccine although it may not keep me from becoming infected with the virus!”  “Being that I don’t have children and considering I had already received my vaccine prior to hearing the infertility rumors that were so-called caused by the COVID-19 vaccine….I panicked. I am so grateful for this information and that I was able to do some research of my own. It definitely helped to ease my mind.” |
| --- | --- | --- |
