# Supplementary material for: Co-Development of a Web Application (COVID-19 Social Site) for Long-Term Care Workers (“Something for Us”): User-Centered Design and Participatory Research Study
Source: J Med Internet Res. 2022 Sep 22;24(9):e38359. doi: 10.2196/38359 (PMC9506501; doi:10.2196/38359)
Supplement: Multimedia Appendix 11 [file jmir_v24i9e38359_app11.docx]

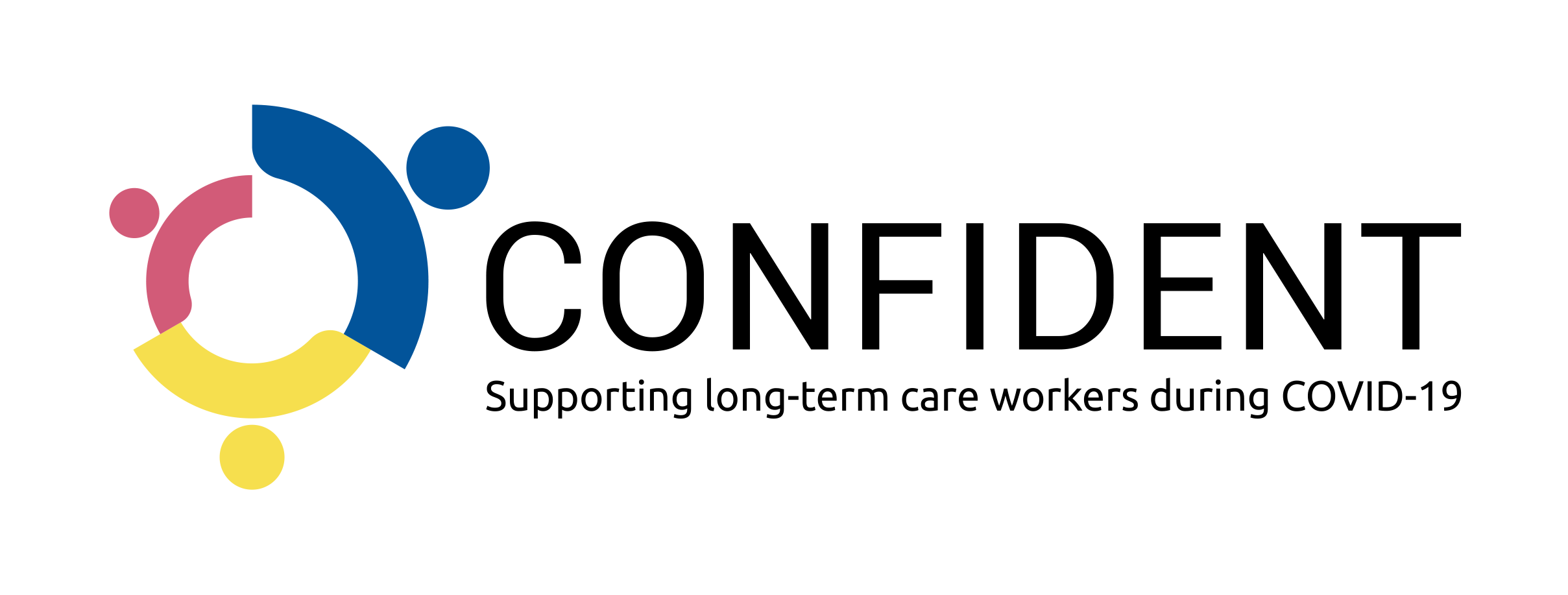


**COVID-19: Master Facts List**

This is a live document, meaning it will be regularly updated as we continue to get new data on the COVID-19 pandemic and the vaccines for it. The facts below have been summarized from those in [this spreadsheet](https://docs.google.com/spreadsheets/d/17yhlNxA-LrupWGpQBb0z9hHVpda8Al0z1gAKOOFGEVM/edit?usp=sharing). Please refer to the full version for more detail, along with the data sources for each fact.

All recent updates will be highlighted in yellow. The dates for when the data sources were originally published are in [pink]. Each fact is accompanied by a title in **blue** to make the document easier to search via keywords.

There is also a table of contents linking to each section that can be accessed either by selecting “Show document outline” from the “View” menu above, or by clicking the small menu icon on the left hand side of the page, just underneath the ruler.

**
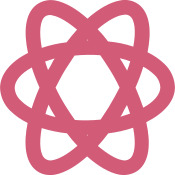
 How bad is COVID-19?
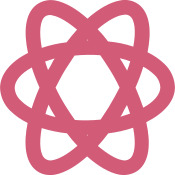
**

## ***General***

#### **Impact of COVID-19 on treatment for other conditions** [Dec. 2021]

- People are not receiving necessary treatment for non-COVID-19 diseases and illnesses because hospital resources are overwhelmed as a result of the pandemic

**COVID-19 hospitalization costs** [Dec. 2021]

- Average cost of being hospitalized with COVID-19 is $20,000
  - No data on how much of this is paid out-of-pocket, but on average $1,300 for pneumonia, as a roughly comparable condition

**COVID-19 linked to changes in brain** [Mar. 2022]

- Significant differences found between MRI scans before and after COVID-19 infection, even with mild cases
  - Overall brain size in infected participants had shrunk between 0.2 and 2%
  - Losses in gray matter in areas linked to smell and to memory
  - Those who had recently recovered from COVID found it a bit harder to perform complex mental tasks
- Researchers uncertain whether these changes are reversible

**Effects of COVID-19 on brain function** [Aug. 2021]

- Brain imaging study of COVID-19 patients showing deterioration of sense of taste and smell, cognitive function, and memory compared to control group without it

**Neurological and psychiatric outcomes after COVID-19 infection** [May 2021]

- Over 200,000 patients who had contracted COVID-19 were observed 6 months after their initial diagnoses of COVID-19, and over 30% were diagnosed with either a neurological or psychiatric illness
- These illnesses included but were not limited to the following
  - Ischaemic stroke
  - Intracranial hemorrhage
  - Parkinsonism
  - Guillain-Barré syndrome
  - Nerve, nerve root, and plexus disorders
  - Myoneural junction and muscle disease
  - Encephalitis
  - Dementia
  - Psychosis, substance use disorder, and mood and anxiety disorders
  - Insomnia

## ***COVID-19 deaths***

##### **Global COVID-19 deaths** [Jan. 2022]

- Around 5.5 million confirmed deaths

**Healthcare worker deaths** [Oct. 2021]

- Over 115,500 healthcare worker deaths from COVID-19 from Jan. 2020 to May 2021

**Mostly unvaccinated dying or hospitalized** [May 2021]

- Of a group of over 18,000 people who died from COVID-19, only about 150 were fully vaccinated, so less than 1%
- Of a group of over 853,000 people hospitalized for COVID-19, only about 1,200 were fully vaccinated (“breakthrough infections”), so around 0.1%

## ***COVID-19 variants***

**Vaccines do not cause variants** [Dec. 2021]

- COVID-19 vaccines do not create or cause new variants of the virus, and instead can actually help prevent new variants from emerging

## ***Infection rates***

**Global COVID-19 cases** [Jan. 2022]

- Around 306 million confirmed cases

**Rising COVID-19 cases during winter** [Dec. 2021]

- National 7-day average for new cases has risen above 120,000 new cases per day, which is a 22% increase from the last 2 weeks
- Hospitalizations increasing by about 20% since the end of November

## ***Pre-existing conditions***

**Most with underlying immune conditions can get COVID-19 vaccines** [Dec. 2021]

- CDC stating that most people with autoimmune conditions can safely get vaccinated against COVID-19

**COVID-19 vaccines less effective in immunocompromised people** [Nov. 2021]

- 2 doses of either Pfizer or Moderna vaccines were 77% effective against COVID-19 related hospitalization for immunocompromised people
- Vaccination still provides a significant degree of protection, but far lower than the benefit to people without immune deficiencies

**
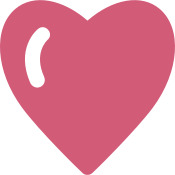
 What are the vaccine benefits?
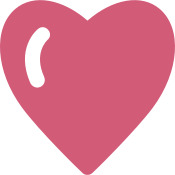
**

## ***General***

**Vaccine efficacy vs. effectiveness** [Nov. 2021]

- “Efficacy” refers to a clinically measurable result acquired in ideal or controlled conditions, such as in a clinical trials
  - For example, trial participants may be carefully chosen or given specific instructions to reduce their risk of infection
- “Effectiveness” is used when the result is acquired under normal circumstances in the real world, which exists outside of the carefully controlled clinical trial setting
  - These numbers are harder to predict and will often be lower than the same number for efficacy

**Choice of COVID-19 vaccine** [Jan. 2022]

- For adults (ages 18 and older), the mRNA COVID-19 vaccines (Pfizer-BioNTech or Moderna) are preferred over Johnson & Johnson’s Janssen COVID-19 vaccine

**Reasoning for vaccinating children** [Dec. 2021]

- Vaccinating children can help protect family members, including those who themselves are not eligible for vaccination
- The vaccines can also help keep children from getting seriously ill even if they do get COVID-19, which will allow them to stay in school or participate in sports, playdates, and other group activities

**COVID-19 vaccines protecting against other viruses** [Apr. 2022]

- T-cell immune responses elicited or enhanced by COVID-19 mRNA vaccines may be able to control SARS-CoV-2 variants and lead to protection against some strains of the common cold and other endemic viruses in the coronavirus family

## ***Preventing death and illness***

**COVID-19 hospitalizations are mostly unvaccinated people** [Jul. 2021]

- People with compromised immune systems and those unvaccinated against COVID-19 make up the majority of patients who end up in the hospital from COVID-19

**COVID-19 hospitalization rates lower for vaccinated people** [Jan. 2021]

- The hospitalization rate is substantially lower for those vaccinated against COVID-19 vs. those who are unvaccinated
- Although weekly rates vary, the cumulative rates of COVID-19 associated hospitalization is several times higher for unvaccinated people across different age groups
  - All adults 18 and older, 13 times higher
  - Adolescents ages 12-17, 10 times higher
  - Adults ages 18-49, 17 times higher
  - Adults ages 50-64, 16 times higher
  - Adults ages 65 and older, 12 times higher

**Pfizer vaccine preventing COVID-19 hospitalization in teenagers** [Oct. 2021]

- A recent CDC-supported evaluation found that 2 doses of the Pfizer vaccine was 93% effective at preventing COVID-19 hospitalization in those ages 12 to 18

**Effectiveness of different COVID-19 vaccines against hospitalization** [Sept. 2021]

- Among U.S. adults without immunocompromising conditions, vaccine effectiveness against COVID-19 hospitalization from March 11 to August 15, 2021 was higher for the Moderna vaccine (93%) than the Pfizer vaccine (88%) and the J&J vaccine (71%)

**COVID-19 vaccination reducing hospitalization and long-term symptoms** [Sept. 2021]

- COVID-19 vaccination (compared with no vaccination) was associated with reduced odds of hospitalization or having more than 5 symptoms in the first week of illness following the first or second dose, and long-duration (past 28 days) symptoms following the second dose
- Almost all symptoms were reported less frequently in infected vaccinated individuals than in infected unvaccinated individuals, and vaccinated participants were more likely to be completely asymptomatic, especially if they were 60 years or older

**COVID-19 illness milder in vaccinated vs. unvaccinated** [Aug. 2021]

- Even among the relatively uncommon breakthrough cases of COVID-19 among the fully or partially vaccinated, those who are vaccinated are more likely to have a milder and shorter illness compared to those who are unvaccinated

## ***Lowering infection rates***

**Herd immunity against COVID-19** [Aug. 2021]

- Herd immunity can be reached when enough people have been vaccinated against a disease and have developed protective antibodies against future infection
- Herd immunity makes it possible to protect the population from a disease, including those who can't be vaccinated, such as newborns or those who have compromised immune systems
  - Using the concept of herd immunity, vaccines have successfully controlled contagious diseases such as smallpox, polio, diphtheria, rubella, etc.
- Unlike the natural infection method in which someone’s immunity against COVID-19 increases after initially contracting it, vaccines create immunity without causing illness or resulting complications

**mRNA vaccines reducing risk of COVID-19 infection** [Jun. 2021]

- A new CDC study finds the mRNA COVID-19 vaccines by Pfizer and Moderna reduce the risk of infection by 91% for fully vaccinated people
  - This adds to the growing body of real-world evidence of their effectiveness

**mRNA vaccines reducing risk of COVID-19 infection** [Mar. 2021]

- Study looking at the effectiveness of Pfizer and Moderna mRNA vaccines in preventing COVID-19 infections among 3,950 study participants in six states over a 13-week period from December 14, 2020 to March 13, 2021
- Results showed that following the second dose of vaccine (the recommended number of doses), the risk of infection was reduced by 90% two or more weeks after vaccination
- Following a single dose of either vaccine, the participants’ risk of infection was reduced by 80% two or more weeks after vaccination

**Effectiveness of Pfizer vaccine in adolescents** [Nov. 2021]

- Results from a longer-term analysis of the safety and efficacy of the Pfizer COVID-19 vaccine in individuals 12 to 15 years of age show that in the pivotal Phase 3 trial, a two-dose series of the Pfizer COVID-19 vaccine was 100% effective against COVID-19, measured 7 days through over 4 months after the second dose

**Effectiveness and safety of Pfizer vaccine in children** [Nov. 2021]

- 90.7% efficacy observed for the 5 to 12 year old group
- Pediatric data indicating the Pfizer vaccine meets all safety expectations for follow up durations and subject number

**COVID-19 vaccination during pregnancy protecting babies** [Jun. 2021]

- Receiving an mRNA vaccine causes the body to produce antibodies against the virus
- In the case of pregnant women who have been vaccinated, these antibodies have been detected in umbilical cord blood, which means that vaccination during pregnancy may also help protect babies against COVID-19

## ***Virus transmission***

**Vaccinated people with COVID-19 are contagious for fewer days** [Oct. 2021]

- The CDC found that the amount of viral genetic material is likely to decrease faster in those vaccinated in comparison to those who are not vaccinated

## ***Booster shots***

**Need for a COVID-19 booster shot** [Dec. 2021]

- Booster shots are recommended because the effectiveness of COVID-19 vaccines wanes over time, which is particularly important as new variants continue emerging

**COVID-19 booster shots increasing protection** [Oct. 2021]

- Israeli data showing booster doses improve protection against COVID-19 infection by 90% compared to just a normal second dose for age groups 16 and older

**COVID-19 booster shots increasing immune response** [Dec. 2021]

- Data from clinical trials showed that a booster shot increased the immune response in trial participants who finished a Pfizer or Moderna primary series 6 months earlier, or who received a Johnson & Johnson single-dose vaccine 2 months earlier
- With this increased immune response, those who receive booster shots will have improved protection against getting infected with COVID-19, as well as COVID-19 symptoms if they are infected

**COVID-19 booster shots protecting against Omicron** [Oct. 2021]

- COVID-19 booster shots significantly restore protection against mild disease caused by the Omicron variant
- After booster doses of the Pfizer vaccine, there was around 70% protection against symptomatic infection for those who initially received the AstraZeneca vaccine, and around 75% protection for those who received Pfizer

**Second J&J dose increasing antibody levels** [Sept. 2021]

- Johnson & Johnson found that a second shot of the J&J vaccine increased COVID-19 antibody levels by 4-6 times in comparison to a single shot

## ***Vaccines vs. natural immunity***

**Natural immunity against COVID-19** [Nov. 2021]

- Getting COVID-19 may offer some natural protection, known as immunity
- Current evidence suggests that reinfection with the virus that causes COVID-19 is uncommon in the 90 days after initial infection
- However, experts don’t know for sure how long this protection lasts, and the risk of severe illness and death from COVID-19 far outweighs any benefits of natural immunity

**COVID-19 vaccines provide more protection than natural immunity** [Aug. 2021]

- A study in Kentucky among people who were previously infected with COVID-19 shows that unvaccinated individuals are more than twice as likely to be reinfected with COVID-19 than those who were fully vaccinated after initially contracting the virus
- Those who were unvaccinated were twice as likely to get reinfected with COVID in comparison to people who were fully vaccinated
- Overall, vaccines offer better protection against COVID-19 than natural immunity alone, and vaccines help prevent reinfection

## ***Immunity over time***

**COVID-19 vaccines still effective** [Nov. 2021]

- While the COVID-19 vaccines’ effectiveness against severe disease and hospitalization has mostly held steady, studies also show that their protection against infection, with or without symptoms, has fallen
- However, public health experts say this decline does not mean that the vaccines are not working, as multiple studies have found that even after several months, the Pfizer and Moderna vaccines remain highly effective at preventing hospitalization

**
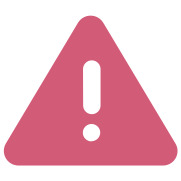
 What are the vaccine risks?
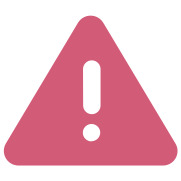
**

## ***General***

**Overall safety of COVID-19 vaccines** [Dec. 2021]

- Over 471 million people in the U.S. have received COVID-19 vaccines
- Results from vaccine safety monitoring are reassuring, with minor common side effects
- Serious safety problems are rare and long-term side effects are unlikely

## ***Side effects***

**COVID-19 vaccines causing mild neurological side effects** [Oct. 2021]

- Neurological adverse events following COVID-19 vaccination are generally mild and resolve themselves quickly
  - This can include side effects like fever and chills, headache, fatigue, myalgia and arthralgia, or local injection site effects like swelling, redness, or pain
- The most devastating neurological post-vaccination complication is cerebral venous sinus thrombosis

**COVID-19 vaccines side-effects in children ages 5-11** [Dec. 2021]

- Children typically experience the same side effects as adults do
  - Soreness, redness, swelling on the arm / injection site
  - Headache, muscle pain, chills, fever, nausea, tiredness
- These usually resolve in a few days, and are a sign that the body is building protection
- Aspirin is not recommended to relieve side effects of vaccination

## ***Long-term risks***

**Long-term safety of COVID-19 vaccines** [Jul. 2021]

- Unlike medications that people take every day that lead to long-term problems as the levels of the drug build up in the body over time, vaccines are designed to deliver a payload and are then quickly eliminated by the body
  - mRNA vaccines degrade especially quickly, and have never been associated with any long-term side effects
- In almost all cases, including COVID-19, vaccine side effects are seen within the first 2 months after rollout
- As more people received COVID-19 vaccines over time, rarer side effects such as clotting disorders (6 days to 2 weeks after vaccination) and inflammation of the heart muscle (no more than 1 month after vaccination) have appeared in an extremely small number of cases
  - These risks are outweighed by the known, much higher risks associated with actually contracting COVID-19

## ***Heart problems***

**Incidence of myocarditis in general** [Oct. 2021]

- The CDC estimates that annually, there are 1 to 10 cases of myocarditis out of 100,000 people in the U.S. (0.01% at most)

**Incidence of myocarditis after COVID-19 vaccination** [Dec. 2021]

- Only around 1.4 in 10,000, or 0.014% of people develop myocarditis (inflammation of the heart muscle) after getting vaccinated against COVID-19

**Risk of myocarditis from COVID-19 vaccination vs. COVID-19 infection** [Sept. 2021]

- A study from a large Israeli healthcare organization found that while COVID-19 vaccines are associated with a higher risk of myocarditis, the risk of myocarditis after a COVID-19 vaccine is much higher (2.7 vs. 11.0 events per 100,000 people)

**Nature of myocarditis after COVID-19 vaccination** [Nov. 2021]

- Myocarditis has been reported after COVID-19 vaccination, especially in male adolescents and young adults
- This is more often after the second dose, and usually within a week of vaccination
- Most patients with myocarditis or pericarditis who received care respond well to medicine and recover quickly, patients can usually return to normal daily activities after their symptoms improve

**Recovery after COVID-19 vaccine related myocarditis** [Nov. 2021]

- COVID-19 vaccine related myocarditis is not the same as the classic, viral myocarditis
- In a CDC study monitoring patients with myocarditis after COVID-19 vaccination, 91% of cardiologists or healthcare providers indicated the patient was fully or probably recovered after 3 months

## ***Serious reactions***

**Lack of increased overall mortality among COVID-19 vaccine recipients** [Oct. 2021]

- From Dec. 2020 to Jul. 2021, vaccine recipients had lower rates of non–COVID-19 mortality than did unvaccinated people after adjusting for age, sex, race and ethnicity, and study site

**Deaths caused by Johnson & Johnson vaccine** [Dec. 2021]

- The CDC has identified 9 deaths that have been caused by or were directly attributed to Vaccine-Induced Immune Thrombocytopenia and Thrombosis (VITT) following the Johnson & Johnson COVID-19 vaccine
- Women ages 30-49 should be especially aware of the increased risk of this rare adverse event, as there are other COVID-19 vaccine options available for which this risk has not been observed

**Risk of Vaccine-Induced Immune Thrombocytopenia and Thrombosis (VITT) after COVID-19 vaccines** [Dec. 2021]

- Vaccine-Induced Immune Thrombocytopenia and Thrombosis (VITT) is a serious adverse event that causes blood clots in large blood vessels
- VITT after Johnson & Johnson’s Janssen COVID-19 vaccination is rare
  - As of December 16, 2021, more than 17.2 million doses of the Janssen COVID-19 vaccine have been given in the U.S.
  - The CDC and FDA identified 57 confirmed reports of people who received the Janssen vaccine and later developed TTS
- Only 3 confirmed cases of TTS following mRNA COVID-19 vaccination (Pfizer, Moderna) have been reported to VAERS (Vaccine Adverse Event Reporting System) after more than 470 million doses of mRNA COVID-19 vaccines administered in the U.S.
  - Based on available data, there is not an increased risk for TTS after mRNA COVID-19 vaccination

## ***Fertility issues***

**No evidence of link between COVID-19 vaccine and fertility** [Dec. 2021]

- COVID-19 vaccines are recommended for women who are trying to get pregnant now or who might become pregnant in the future, as well as their partners
- There is currently no evidence that any vaccines, including COVID-19 vaccines cause fertility problems in women or men

**Effects of COVID-19 vaccine on menstrual cycles** [Jan. 2022]

- The vaccines for COVID-19 are associated with small changes in menstrual cycle length but not the length of actual menstruation, or duration of bleeding

## ***VAERS***

**Vaccine Adverse Event Reporting System** [Nov. 2021]

- After clinical trials, the VAERS is how the CDC monitors vaccine safety
  - It is set up to capture potential adverse events caused by vaccines
  - The VAERS cannot determine whether a vaccine actually caused something, only that the adverse event occurred at a point in time after vaccination
- The FDA requires healthcare providers to report any serious adverse event, including death, that happens after COVID-19 vaccination, regardless of whether they think the vaccine caused the event
  - Many deaths do occur coincidentally after vaccination, and even if a vaccinated person drowns or is struck by lightning, the death must be reported to the VAERS
- The true number of deaths currently attributed to COVID-19 vaccines in detailed scientific investigation is extremely small
  - The CDC has identified only 9 deaths causally associated with COVID-19 vaccination, specifically the Johnson & Johnson vaccine

**
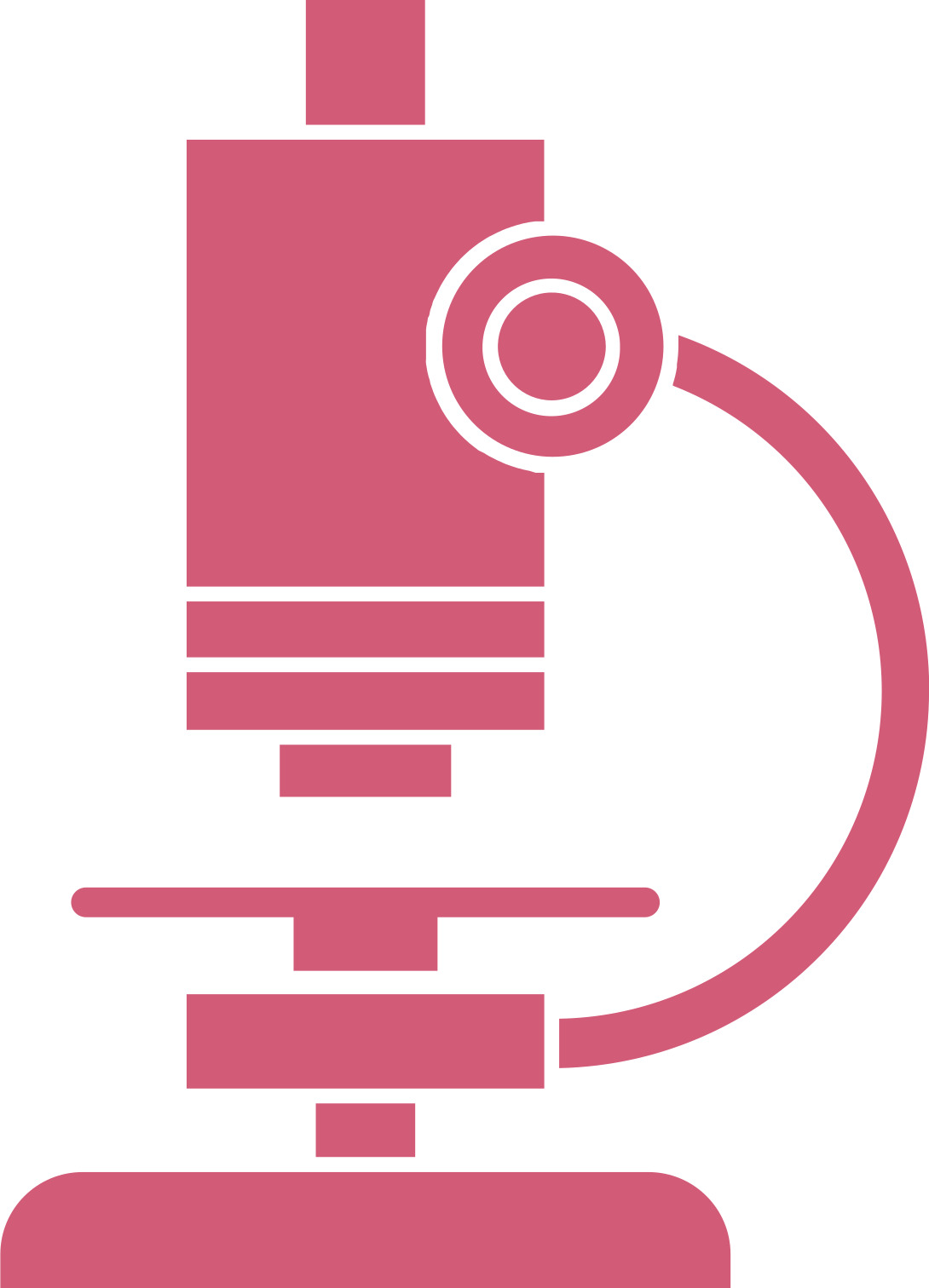
 How are the vaccines made?
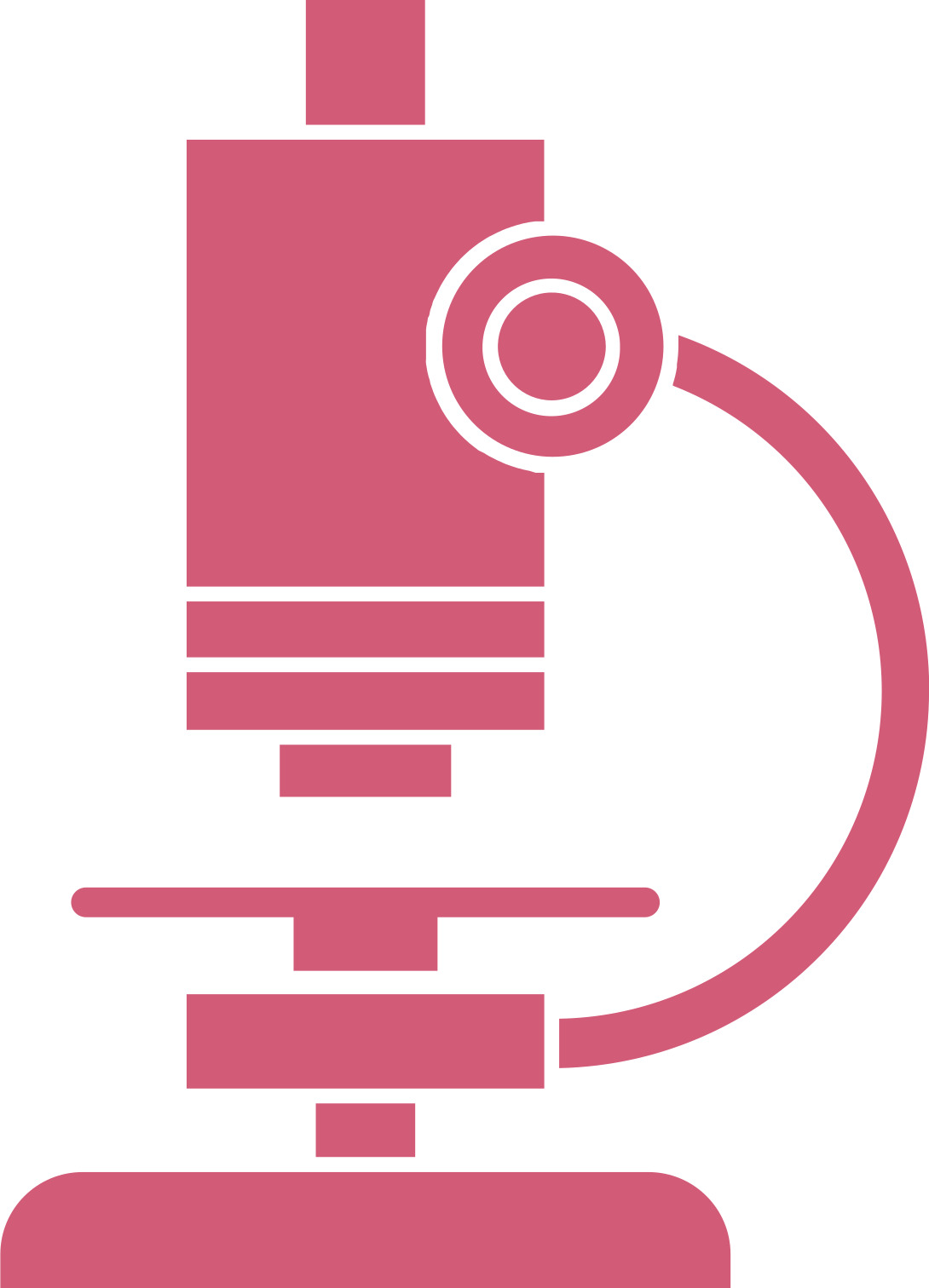
**

## ***General***

**COVID vaccine costs** [Oct. 2021]

- Pricing varies depending on the country and company
- Currently, all COVID-19 vaccines are free to anyone in the U.S. and are being paid for by insurance companies and the government
  - Moderna, $15 per dose
  - Pfizer, $24 per dose
  - J&J, $10 per dose, not for profit

**How mRNA vaccines work** [Nov. 2021]

- mRNA enters the muscle cells and instructs the cells’ machinery to produce spike proteins, after which the cells break down the mRNA and remove it
- Cells then display the spike protein pierce on their surfaces, and the immune system recognizes that the protein doesn’t belong there
- The immune system is then triggered to produce antibodies and activate other immune cells to fight off what it believes is an infection
- At the end of the process, the body will have learned how to protect against future infection from the virus that causes COVID-19

**COVID-19 is not injected into the body** [Nov. 2021]

- The COVID-19 vaccine does not inject the virus into an individual’s body
- It is an mRNA vaccine, which means that it instead injects mRNA, which will enter the cells and instruct to the body to produce spike proteins
- People cannot contract COVID-19 from the COVID-19 vaccines

**COVID-19 vaccine dosage for children** [Nov. 2021]

- Unlike many medications, vaccine dosages are based on age at the time of vaccination and not size or weight
- If a child turns from 11 to 12 years of age in between their first and second dose, the second dose should be the Pfizer vaccine for people 12 years and older
- However, if the child receives the Pfizer-BioNTech COVID-19 vaccine for children ages 5 through 11 for their second dose, they do not need to repeat the dose

## ***FDA approval***

**Emergency approval process for COVID-19 vaccines** [Aug. 2021]

- Not every vaccine is granted an emergency use authorization before full approval
  - The FDA authorized 3 vaccines to answer an urgent need to protect people from COVID-19, which was and continues to be a public health emergency
- When it was clear in 2020 that COVID-19 was a rapidly spreading, dangerous disease, the FDA worked with vaccine manufacturers, independent laboratories, academic research centers, and nonprofit organizations to quickly gather and interpret large amounts of data on the vaccines
- The data showed the vaccines were very effective in preventing severe COVID-19 and the risks of serious side effects was extremely low
  - Based on these findings, emergency use approval was granted by the FDA so that people could be vaccinated earlier and be protected from the serious effects of COVID-19
- Full approval is granted when, over time, the FDA has amassed even more scientific evidence to support use of the COVID-19 vaccines, showing that the benefits of the vaccine are greater than its risks, and that the vaccines can be manufactured reliably, safely and with consistent quality

## ***Speed of development***

**Rapid development of COVID-19 vaccine** [Dec. 2020]

- Decades of past research on mRNA vaccines have allowed scientists to develop the COVID-19 vaccine rapidly relative to some other vaccines, but this does not mean that the process it was rushed
- Moderna previously used mRNA technology to develop vaccines against other diseases prior to the COVID-19 pandemic

## ***Vaccine ingredients***

**Active vs. inactive ingredients** [May 2021]

- Active ingredient is mRNA
- Inactive ingredients are lipids, salts, sugars, acids, and acid stabilizers

**Vaccine components remaining in body** [Nov. 2021]

- mRNA degrades within days, although the spike proteins may stay in the body for up to a few weeks

## ***Booster shots***

**Need for booster shots in general** [Aug. 2018]

- Vaccines requiring a booster shot is not uncommon, as studies have shown that more than one dose is often needed to allow a strong immune response to develop
- Some vaccines require additional doses because immunity can decrease over time after initial vaccination

**FDA booster shot guidance** [Dec. 2021]

- The FDA has authorized three COVID-19 vaccine boosters (Pfizer, Moderna, Johnson & Johnson) and determined it is safe to get a booster or second dose that is a different brand than the initial dose or doses
- Immunosuppressed people who originally received the Pfizer or Moderna vaccines can get a booster shot after it has been at least 28 days since their last dose
